# The role of single protein elasticity in mechanobiology

**DOI:** 10.1038/s41578-022-00488-z

**Published:** 2022-10-24

**Authors:** Amy EM Beedle, Sergi Garcia-Manyes

**Affiliations:** 1 Department of Physics, Randall Centre for Cell and Molecular Biophysics, Centre for the Physical Science of Life and London Centre for Nanotechnology, King’s College London, Strand, WC2R 2LS London, United Kingdom; 2 Institute for Bioengineering of Catalonia (IBEC), the Barcelona Institute of Technology (BIST), 08028 Barcelona, Spain; 3 Single Molecule Mechanobiology Laboratory, The Francis Crick Institute, 1 Midland Road, London NW1 1AT, London, UK

## Abstract

In addition to biochemical signals and genetic considerations, mechanical forces are rapidly emerging as a master regulator of human physiology. Yet the molecular mechanisms that regulate force-induced functionalities across a wide range of scales, encompassing the cell, tissue or organ levels, are comparatively not so well understood. With the advent, development and refining of single molecule nanomechanical techniques, enabling to exquisitely probe the conformational dynamics of individual proteins under the effect of a calibrated force, we have begun to acquire a comprehensive knowledge on the rich plethora of physicochemical principles that regulate the elasticity of single proteins. Here we review the major advances underpinning our current understanding of how the elasticity of single proteins regulates mechanosensing and mechanotransduction. We discuss the present limitations and future challenges of such a prolific and burgeoning field.

## Introduction

Mechanical forces are intricately and inexorably related to life^1^, and regulate innumerable biologically processes including development and disease^2–5^. Mechanistically, transient forces sensed at the cell membrane rapidly reach the cytoskeleton and eventually the nucleus following a myriad of complex and cell- and time-dependent mechanisms^1,6–14^. From a quantitative perspective, a suite of techniques is now available to measure forces at the cellular^15^ and tissue^16^ level, providing an increasingly precise knowledge of the different mechanosensing and mechanotransduction pathways employed by eukaryotic cells. Yet a molecular understanding of these cellular- and tissue-scale phenomena has fallen behind, in part because of the large number of proteins at play, and also due to the technical complexity of measuring the mechanically-regulated conformational dynamics of each individual protein, most of them bearing forces as low as a few picoNewtons.

New evidences are revealing a significant number of proteins that are physiologically exposed to mechanical forces^17^. Proteins have evolved a plethora of sophisticated nanoarchitecture designs, where the presence of a few strategically-placed chemical bonds within their structure ultimately provides a rich repertoire of mechanical functionalities. Put simply, the mechanical stability of proteins can be easily understood in terms of the nature and number of the sticky chemical bonds encountered by the propagating force as it crosses the protein’s backbone. Consequently, modifying the force-propagation route can result in a change in the protein’s mechanical resistance. Upon unfolding, proteins reach stretched conformations that expose to the solvent residues that were previously hidden in the compact native state^18^, enabling new chemical reactivity or protein binding that might initiate mechanotransduction pathways. To recover their mechanical physiological function, proteins need to refold back to the mechanically rigid native state upon a concomitant force reduction.

Here, we will review the major discoveries underpinning the field of single protein mechanics, focusing on how the intrinsic elasticity of proteins directly impacts mechanobiology. As such, the review will not cover the important discoveries describing the general mechanisms governing mechanical protein (un)folding and the underlying energy landscape^19–28^, or the dynamics of biological (or synthetic) polymers under force^29^. Similarly, we will not discuss the functioning of cellular molecular motors^30^. Finally, those looking for in-depth technical comparative information of the different single molecule mechanics techniques might want to complement the information provided here with specifically dedicated reviews^31–34^.

While many fascinating force-bearing molecular mechanisms have been recently discovered in bacteria —including for example the outstanding high tensile forces that *Staphylococcal* adhesins are able to resist^35,36^, often underpinning catch bond behaviour, the large mechanical forces that keep the cohesin-dockerin complex together in cellulolytic bacteria^37,38^, or the also seemingly large forces between staphylococcal surface proteins and their extracellular ligands^39–41^ —this review is eminently focused on the nanomechanical properties of those proteins directly involved in the mechanosensing and mechanotransduction of eukaryotic cells. The present review is organised such that proteins involved in particular cellular functions, or embedded within the same cellular region, are grouped together (regardles of the technique used to study them). The final passages of the review considers novel and challenging experiments aiming at uncovering the mechanical role of individual proteins when placed in the cellular context. We will conclude by exposing what are, in our view, the most stimulating next challenges in the field.

## Measuring protein nanomechanics at the single molecule level

**Figure 1:**
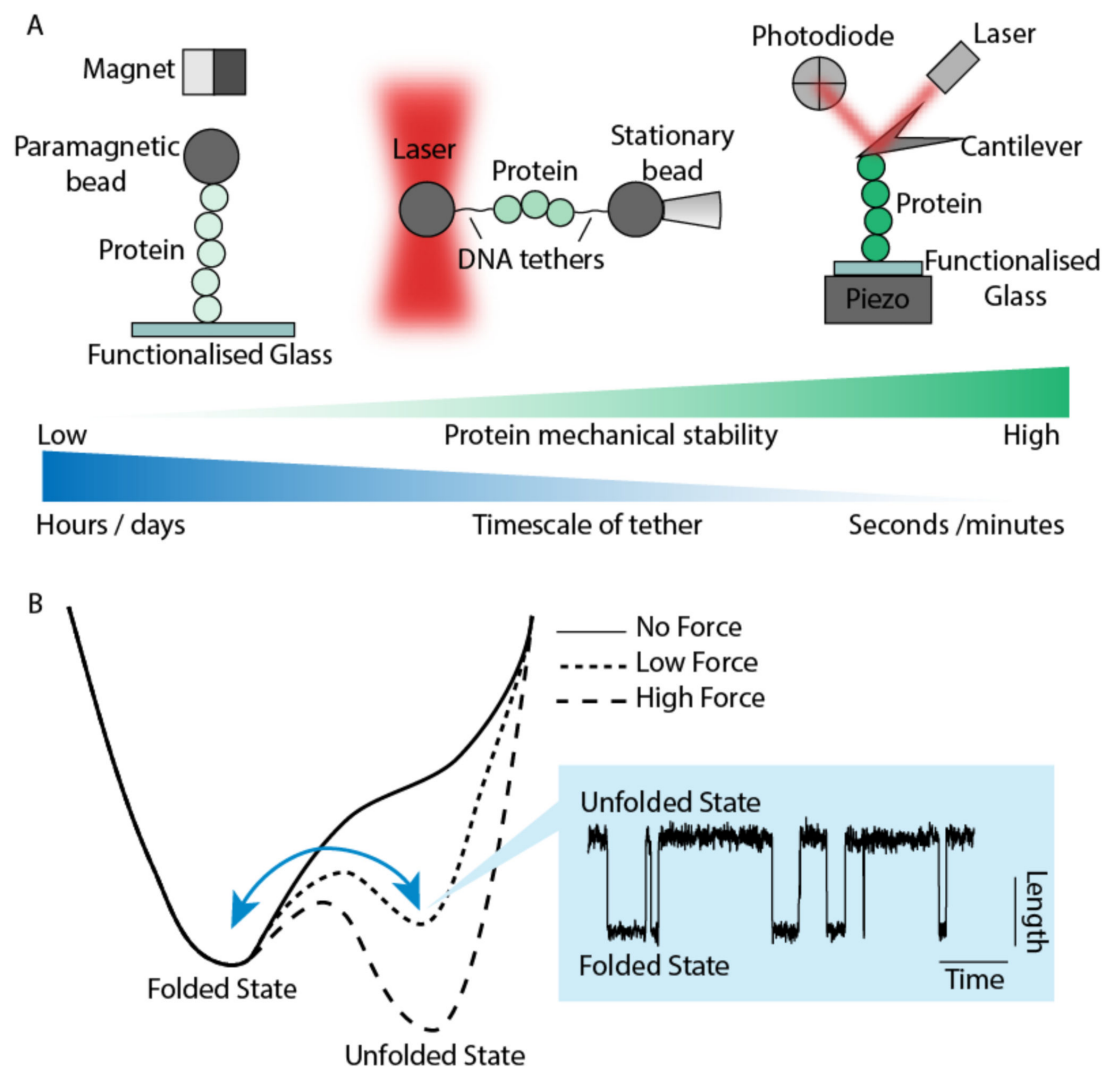
Single molecule force spectroscopy techniques enable the mapping the (un)folding energy landscape of a protein under force. The main force spectroscopy techniques rely on an individual molecule tethered between one fixed surface and a second surface, the position of which is precisely controlled. The adequacy of each technique depends on the mechanical properties of the studied protein and the desired sampling time. For example, magnetic tweezers are ideally suited to investigate the (un)folding dynamics of mechanically weak proteins over long times, up to hours or even days. At the other end of the spectrum, the AFM is ideally suited to examine high mechanical stability proteins, and experiments typically remain stable for a few seconds to minutes. (A) In a magnetic tweezers experiment, a molecule is attached at one end to a paramagnetic bead. The force applied to the bead is controlled by the localization of a pair of permanent magnets mounted above the surface (typically mounted on a voice coil or a piezoelectric actuator). Optical tweezers trap a bead in a laser beam which can both apply or sense movement. In a typical scenario, the protein is tethered between two long DNA handles that are, in turn, attached to the trapped (or fixed) beads. In the AFM, a molecule is tethered between a piezoelectric actuator and a flexible Hookean cantilever. Mechanical force is applied when the piezo retracts away from the cantilever, and the mechanical response of the molecule is measured by monitoring the deflection of a cantilever. (B) The application of mechanical force to the protein reduces the height of the energy barrier between the folded and the unfolded states. In the simplest approximation, the Bell/Arrhenius model predicts an exponential acceleration of the unfolding rate (α_u_) with the applied force (*F*), and indicates that the height of the energy barrier (Δ*E*) is effectively reduced by a factor -*F*Δ*x*. The proportionality factor, Δ*x*, is a direct indication of how sensitive (brittle) each protein is to force denaturation. If the applied force is high enough, the molecule will traverse the energy landscape and remain in the energetically-favorable unfolded state. However, if the force is low enough such that the unfolded and folded state are energetically similar, the protein can hop between the folded and unfolded state in equilibrium at that particular force.

Three main single molecule nanomechanical techniques have provided the majority of the available knowledge on protein mechanics, namely the force spectroscopy modes of atomic force microscopy (AFM)^31^, optical tweezers^42^ and magnetic tweezers (smMT)^32^, Figure 1. Given their different operational principles and their distinct optimal range of operational forces, the three techniques are highly complementary. In brief, single molecule AFM is based on a Hookean bending cantilever that applies forces to individual biomolecules and is generally suited to explore proteins with high mechanical stabilities (∼30 pN to several nN)^31^. Despite several recent technological improvements^43^, the intrinsic cantilever fluctuations typically mask the behaviour of proteins at lower forces. By contrast, optical tweezers are suited to proteins of relatively low mechanical stabilities (∼3-60 pN), which are bracketed between long DNA handles^44^, in turn attached to optically-trapped beads^26^. Both AFM and optical tweezers can work under force-extension conditions – measuring the dynamically changing restoring force experienced by the protein as it is stretched under constant velocity – or under force-clamp conditions, where the protein is held at a constant force and its extension is measured over time. SmMT applies forces by a permanent magnet to an individual protein that is tethered between a glass surface and a paramagnetic bead. Given that the magnetic field decays slowly with the distance, the applied force can be considered constant within short (nm) distances and therefore effectively work under passive force-clamp conditions^45,46^, significantly increasing the stability of the measurement over the two previous techniques. Concomitant to the development of different experimental techniques, the field has benefitted from steered molecular dynamics (SMD), whereby proteins are stretched in silico^47^. Those computer experiments have overall provided a detailed atomistic perspective on the (sub)molecular mechanisms that underpin the distribution of molecular forces across the protein backbone^48^.

## The energy landscape of proteins under force

Using force as a denaturant requires a quantitative formalism to evaluate how the highly-localised and directional mechanical stress modifies the energy landscape of a folding protein. A very (over)simplified description^49^ considers the overall protein’s energy as the result of the addition of two energy terms: (i) an enthalpic component that underpins the low-energy, protein’s distinctive native state, and (ii) an entropic component that captures the behaviour of *any* polymer under force and that can be well-described by models of polymer elasticity such as the Worm-like chain (WLC) or the Freely-Jointed Chain (FJC)^50,51^.

When proteins are mechanically denatured, the application of force along a 1D across the protein backbone will disrupt a series of key enthalpic interactions (namely hydrogen bonds and hydrophobic interactions) that define the protein’s native state. Quantification of the life-time resistance to force of these ‘sticky bonds’ defines the mean passage time for mechanical unfolding, enabling calculation of the height of the main barrier determining unfolding. Theoretical approaches combined with experimental observations provide a first description of how the barrier’s shape is deformed by force^52^. Once unfolded, the protein’s end-to-end average length can be accurately described by the WLC or FJC models of polymer elasticity, and the equilibrium position at each force can be experimentally measured by noise fluctuations^53^. While this description generally applies to all proteins, what are the microscopic mechanisms that help shaping the subtleties defining the (mechanical) energy landscape for each particular protein?

## Molecular mechanisms regulating protein nanomechanics

Through the years, a plethora of mechanisms at the molecular level have emerged as effective regulators of the mechanical stability of the protein’s native state^45^, namely (i) topology; as a rule of thumb, β-sheet proteins with antiparallel strands tend to be mechanically stable, whereas proteins rich in α-helix content are normally mechanically labile. (ii) Mutations, especially in the mechanical clamp, are also able to tailor the overall protein mechanical stability^54^. (iii) The pulling direction determines mechanical stability, underpinning the vectorial and anisotropic nature of the denaturing force^55,56^. (iv) Chemical modifications such as the formation of reversible disulfide bonds or irreversible isopeptide bonds can shortcut the protein, drastically changing its stiffness^57–59^. (v) Upon protein unfolding, previously cryptic residues become exposed to the solvent^18^, and can become chemically reactive^60^ and susceptible to new ligand binding^61^. The modulation of the protein’s mechanical stability will determine its unfolding probability, with direct knock-on effects on mechanotransduction.

## Dynamic mechanical (un)folding regulates mechanotransduction

Mechanical unfolding softens the protein, transforming the mechanically stiff native form (with an associated persistence length *p* ∼ 4-5 nm) into a mechanically compliant unfolded and extended state (*p* ∼ 0.4nm)^62^. It is enticing that such a conformationally-mediated change in stiffness might underpin important mechanotransduction events. Folded and stiff domains propagate mechanical signals efficiently. However, their large rigidity may result in a sharp force increase, easily leading to the fracture of the mechanically connected proteins. By contrast, soft molecular springs need to massively extend before ‘delivering’ forces to contiguous domains. While this scenario prevents mechanical damage, force propagation is comparatively slow and ineffective, requiring proteins to massively deform at the risk of rendering an unphysiologically compliant tissue. In the intermediate scenario, shock-absorbing proteins guarantee efficient mechanosensing and mechanotransduction through tuning their mechanical unfolding/refolding; when the applied forces are low-to-moderate, proteins would keep in their folded state and propagate forces fast. However, if the sensed forces are too high, protein unfolding will act as a protective mechanism resulting in the storage of the excess of mechanical work. Upon mitigation of the high-force conditions, proteins can collapse and refold back into their native state, returning part of the accumulated heat back to the system. A large number of proteins with a physiological mechanical role are composed of multiple domains in tandem^63^. Given that multidomain proteins have a higher probability to misfold and aggregate^64–66^, why do mechanical proteins typically arrange in tandem repeats^63^? It is tantalizing to consider that nature might have evolved the mechanical architecture of large polyproteins made of a string of independent shock absorbers according to each particular physiologically-relevant context. To ensure an optimal regulation of force propagation while avoiding mechanical damage, proteins might adjust the number of folded and unfolded domains at any given time, based on the distinct (un)folding dynamics of each independent module^28^.

In what follows we review the large body of literature collectively uncovered by the force spectroscopy protein nanomechanics field, where changes in protein elasticity underpin function. We have systematically explored the nanomechanics of these proteins according to their sequential position within the long cellular mechanotransduction pathway, from the cell surface to the nucleus.

## Nanomechanical regulation of the focal adhesion complex - the paradigmatic role of talin

The extracellular matrix (ECM) is a network of multidomain proteins (including collagen, elastin, fibronectin, tenascin and laminin) and polysaccharides that assemble into structurally stable meshwork that houses cells and contributes to the mechanical properties of tissues^67^.

**Figure 2:**
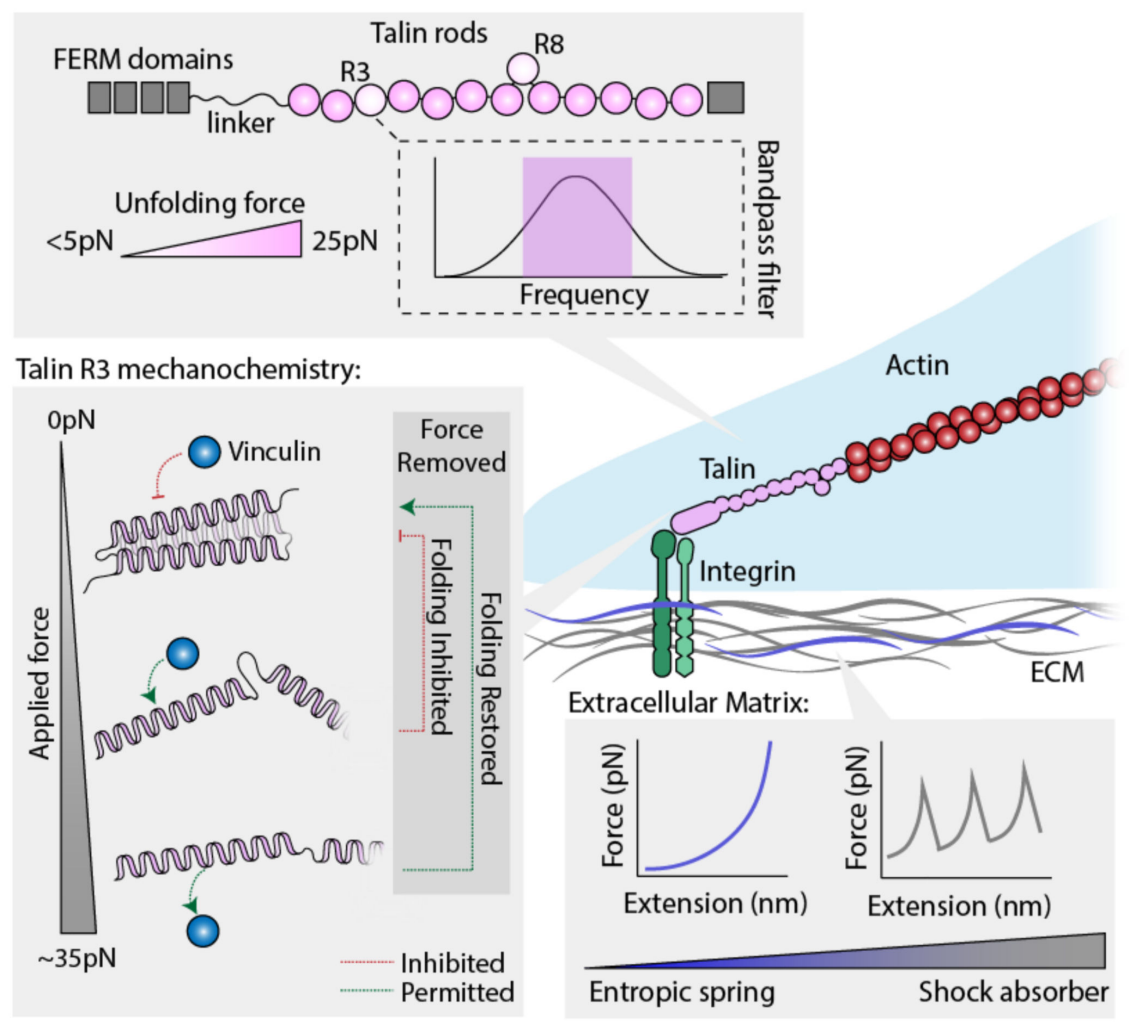
Nanomechanical regulation of the cell matrix and the focal adhesion hub. The mechanical behaviour of the extracellular matrix (ECM) is governed by the mechanical behaviour of two types of molecular constituents. Molecules such as elastin and the polysaccharides readily extend under an applied force, hence behaving as entropic strings. They underpin the reversible elastic properties of the ECM. By contrast, molecules that exhibit significant resistance to mechanical unfolding, such as tenascin and fibronectin, tend to work as (often reversible) shock-absorbers. When streteched under constant velocity conditions, polyproteins that naturally work as shock absorbers display a saw-tooth like force extension profile, where each force peak corresponds to the unfolding and extension of one individual domains. The area under each peak is a direct measurement of the stored heat that is dissipated as the segmented protein is gradually elongated after mechanical unfolding^73^. In particular, talin couples integrins at the cell surface with the actin cytoskeleton. All the talin rod domains are mechanically labile, unfolding between 5-25 pN. The R3 domain – one of the most mechanically vulnerable – functions as a bandpass filter, capable of filtering out mechanical noise and only sensing the average applied force, and is able to bind to vinculin once talin is mechanically unfolded and the previously cryptic binding sites become exposed to the solvent.

The majority of single molecule experiments have focused on understanding the large multidomain proteins fibronectin and tenascin. Characterization of fibronectin type III domains revealed a high mechanical stability, spanning ∼60 – 200 pN for the different domains^68,69^. Similarly, the Fn type III domains of tenascin-C require ∼137 pN to unfold^70^. This high mechanical stability and shock-absorber behaviour is in stark contrast with other major components of the ECM, such as the soft elastin proteins and polysaccharides, exhibiting a mechanical behaviour dominated by entropic extensibility ^71,72^ (Figure 2).

Mechanistically, the mechanical unfolding of fibronectin type III domains has two associated energy barriers. The first corresponds to a rotational movement where the protein β-sheets must align with the direction of the applied force. The second barrier is related to the actual rupture of the key set of hydrogen bonds ^74^. Disrupting the mechanical clamp with the Proline88 mutation decreases the unfolding force from ∼130pN down to <20pN^75^. Conversely, creating stabilizing bonds in the mechanical clamp increases the mechanical stability by ∼30pN^76^. Perhaps most dramatically, by piecing together regions from different proteins it is possible to create a new hybrid protein with specific mechanical properties. Replacing 15 amino acids of the Fn10 domain of fibronectin with the core of the more mechanically stable Fn3 domain of tenascin, gives rise to a modified fibronectin domain with a 20% increase in its mechanical stability, recapitulating the mechanical properties of the tenascin domain^77^. A striking feature of many of these multidomain ECM proteins is their extensive binding sites for other molecules, including cell binding sites, cytokines and other ECM proteins^78^ ^79^. Interestingly, many of these sites are cryptic when the protein is folded, suggesting that force-induced domain unfolding of ECM proteins can act as a functional switch to regulate the accessibility of those otherwise hidden binding sites^80,81^. For example, unfolding of the fibronectin FnIII-1 domain exposes a previously cryptic binding site for self-assembly which leads to the formation of fibronectin fibrils^82^.

These single molecule experiments offer the possibility to rationally design functional ECM-mimicking proteins, hydrogels and biocompatible materials with tuneable mechanical properties ^83^ ^84^, or sensors that report on the mechanical configuration of the tissue components^85^. These advances in the field of ECM mechanobiology highlight the importance of gaining an in-depth single molecule knowledge to scale up our understanding to higher length scales.

The primary mechanism by which cells interact with the ECM is via membrane proteins on the cell surface. In particular, integrins are a family of transmembrane proteins that form heterodimers, with an extracellular domain which binds to various ECM components and an intracellular domain^86^. Seminal cellular experiments where the substrate stiffness was rationally changed revealed that a5b1 integrin binds to the RGD of Fn10, and that, when bonds are tensioned (ie. when plated on stiff substrates), the synergy site is enganged through a catch-bond mechanism^87^. Single molecule force experiments on full-length integrins are still challenging. The present limitations are likely to be circumvented with the use of lipid bicelles^24,88^ or nanodiscs ^89^. Integrin-based adhesion complexes, or focal adhesions (FA), are molecular hubs which assemble and disassemble in response to a wide range of stimuli, including the matrix composition, ligand density, chemical cues, and crucially, mechanical force^90^. This mechano-sensing behaviour is, in part, facilitated by the integrin-talin-actin link, which establishes a physical connection between the external cellular environment and the cell interior^91,92^. However, elucidating how this molecular tether senses mechanical forces that trigger signalling cascades deep inside the cell requires a detailed understanding of the relationship between protein conformation and mechanical stimuli.

Talin binds to integrins at its N-terminus and to F-actin at its C-terminus. It is formed 4 FERM domains, an unstructured region and 13 helical bundles – known as the rod domains – and an actin binding domain^93^. This configuration of folded domains in series is perfectly suited for efficient force transmission since force is distributed equally amongst all domains, causing the mechanically weakest domains to undergo unfolding first (Figure 2). While AFM experiments revealed that all talin domains are mechanically vulnerable to unfolding^94^, SmMT uncovered a detailed description of the mechanical hierarchy of talin^95^. The stability of the rod domains ranges from ∼5 – 25 pN. The two weakest domains (∼5 pN) are the R3 and R8, containing a threonine belt that destabilizes the protein’s hydrophobic core. A four point mutation in the R3 domain (IVVI mutant) increases the mechanical stability up to ∼8 pN^96^. While this suggests that both R3 and R8 unfolding is probable *in vivo*, the R8 domain adopts a unique geometry such that it is shielded from the force by the R7 domain. Therefore, R8 unfolding occurs concomitantly with the unfolding of the more mechanically stable and protective R7 domain at ∼14pN. However, R7 domains in isolation unfold at lower forces of ∼10pN. This suggests a symbiotic relationship, whereby R7 protects R8 from mechanical unfolding, while R8 mechanically stabilizes its R7 neighbour.

To assess whether complex force perturbations (recapitulating the noisy cell environment) affect talin’s (un)folding kinetics, Tapia-Rojo et al used a magnetic tape head to control the magnetic field by an electrical signal, enabling both rapid changes in the force with a bandwidth of 10 kHz and the input of more complex signals^97^. Subjecting the R3 domain of talin to oscillatory force signals revealed that talin dynamics are responsive to a specific frequency range, determined by the mechanical stability of the domain. This behaviour is reminiscent of a band-pass filter, rejecting frequencies outside of an allowed range, and adds a new paradigm to our understanding of the sensitivity of force-dependent protein signalling inside the cell.

For effective mechanotransduction, these force-induced conformational changes in talin must lead to a change in function. The focal adhesion complex is a highly orchestrated hub composed of a multitude of interacting proteins. In particular, talin contains up to 11 vinculin binding sites^98^, however, in the native state many are hidden within the core of the protein. This raises the question, what is the purpose of hidden binding sites? Seminal work mechanically stretching talin in the presence of diffusive vinculin, revealed that mechanical unfolding exposes the vinculin binding site and allows binding^61^. In the R3 domain, this is a highly force-dependent process, where there is a peak in the binding probability at ∼5 pN^99^. This binding event is concomitant with a shortening of the end-to-end length of talin, suggesting that binding of the vinculin head induces a conformational change to talin compatible with a coil-to-helix transition, whereby vinculin must do mechanical work against the pulling force across talin to facilitate a strong protein-protein interaction^99^. However, as the force is further increased, the binding probability sharply decreases, and at forces >30pN vinculin binding is inhibited. In the absence of vinculin, talin folding is a rapid and efficient process, however, upon vinculin binding, talin is locked into an unfolded conformation that is persistent over the timescales of hours. A high force pulse (∼25-40 pN) expels vinculin from the talin polypeptide, by uncoiling and destabilizing the helices and disrupting the preferred vinculin binding configuration, enabling talin to regain refolding capability. Recent experiments with the full-length vinculin diminishes the binding efficiency to talin compared to the vinculin D1 domain^100^.

The relationship between force and protein binding is further complicated when additional talin binding partners are considered. Deleted in liver cancer 1 (DLC1) is a FA protein that binds to the native state of the R8 talin domain^101^ and regulates cell movement and signalling^102^. DLC1 binding does not alter the mechanical stability of R8. However, mechanical unfolding of the R8 domain disrupts the binding interaction between talin and DLC1, ultimately displacing DLC1 from the focal adhesion complex^103^. Alternatively, Cyclin-dependent kinase 1 (CDK1) binds to the folded state of the R7 and R8 domains, and introduces a phosphorylation modification at the end of the R7 domain and close to the point of the R8 insertion. Experiments on a phospho-mimetic mutant of the R7-R8 peptide reveal that phosphorylation weakens the R7-R8 domain interaction by ∼4 pN^104^. Consequently, we have an emerging view indicating that mechanical force serves as a functional switch for talin, modulating structural conformation and thus regulating the various binding partners depending on the magnitude of mechanical force.

We are yet to understand the precise structural configuration of FAs, therefore it is challenging to predict which proteins lie within the force propagation pathway. A first approach is to select proteins that are tethered at both termini, ideally with one end attached to a motile structure, like the contractility network. One such protein is focal adhesion kinase (FAK), which is tethered between the cell membrane and the actin cytoskeleton via paxillin^105^. FAK contains a mechanosensitive phosphorylation site that is activated when cells are seeded on high rigidity substrates^106^. Mechanistically, activation occurs upon disrupting the binding interface between the FERM and kinase domains, which triggers autophosphorylation of FAK at tyrosine397^107^. Therefore, it is highly plausible that mechanical force may trigger this conformational change. Indeed, low mechanical forces (<25pN) measured with AFM are sufficient to trigger FERM-kinase domain separation, and crucially, the domain interface is disrupted prior to domain unfolding^108^.

It is plausible that in the FA there are molecular configurations whereby the binding interface between two proteins is subjected to mechanical force. However, quantifying the stability and lifetime dynamics of protein-protein interactions under force is challenging due to the lack of a well-defined fingerprint (except, for example, with talin’s length change upon vinculin binding and unbinding^99^) as domain rupture can be easily confused with spurious detachment of the protein from the experimental probe. To circumvent this limitation, one possible strategy is to insert a long, unstructured and flexible linker between the two proteins of interest^109^, thus maintaining a connection despite separation, and providing an unambiguous fingerprint for the dynamics of domain unbinding/binding^110^. This strategy was utilized by Le and Yu et al, to probe the interaction between vinculin and a vinculin binding site in talin^111^. Upon application of physiological forces (∼10pN) the binding site remained intact for >1000 seconds. Even with forces as high as ∼25 pN, was the interaction sustained on the timescale of seconds. Alternatively, the KANK family of proteins mediate the interaction between the actin cytoskeleton and microtubules at focal adhesion sites^112^. KANK1 packs against the side of the R7 talin domain, therefore when talin is subjected to force it is likely that the binding site between KANK1 and talin R7 is subjected to force. Consequently, the strength and lifetime of this interaction will determine the degree of connectivity between the microtubule and actin networks^113^. Force-dependent lifetime experiments revealed that the KANK1-R7 interaction undergoes a sharp transition from catch bond behaviour (where bond lifetime increases with increasing force) to slip bond behaviour (where bond lifetime decreases with increasing force) at 6 pN^114^. This catch-to-slip switching defines an optimal force range for the KANK1-R7 interaction, which coincides with the physiological forces in the cell.

Altogether, these results reveal the complex relationship between force, protein conformation and chemical reactivity of a few key focal adhesion proteins, elucidating some of the mechanisms that allow the adhesion complex to respond to a range of physiologically relevant forces. However, given the extraordinary molecular complexity of the FA complex, we are still far from a comprehensive picture.

## The nanomechanics of the cell-cell interactions

To maintain tissue integrity and function as a collective entity, cells must mechanically interact with each other. This connection must endow sufficient strength while providing flexibility to allow the tissue to move and deform. These demanding mechanical requirements must be provided by the underpinning molecular players^115,116^. Cadherins are transmembrane proteins that mediate calcium-dependent cell-cell adhesion in a plethora of tissues^117,118^. The extra-cellular region of cadherins is formed of 5 extracellular domains arranged in tandem and form homotypic interactions with the cadherins on opposing cells, forming tight inter-cellular bonds^119^. In adherens junctions, the intercellular region of cadherins is linked to β-catenin, which links to α-catenin which in turn binds directly to the actin cytoskeleton, or indirectly via adapter proteins such as α-actinin or vinculin.

Structural studies demonstrate that interacting cadherins adopt two distinct structural conformations; an X-dimer, where the extracellular domains form surface interactions^120^, or a strand-swapped dimer, whereby each cadherin inserts a conserved tryptophan into a hydrophobic pocket in the adhesive partner^121^. To understand whether these structural conformations underpin a mechanical function, Rakshit et al performed experiments on specific cadherin mutants that locks the protein complex into one of these two binding conformations^122^. The X-dimer displayed a biphasic mechanical response, functioning as a catch bond up to forces of 30 pN, beyond which it transitions into a slip bond. Mechanistically, the catch bond occurs when tensile forces applied to a calcium-saturated cadherin dimer modify the protein orientation such that new *de-novo* hydrogen bonds form on the N-terminal β-strands on the EC1 domain^123^, increasing the resistance to the applied force. Alternatively, the more mature strand-swapped conformation only exhibits slip-bond behaviour. Catch bond behaviour is abolished in this conformation as the EC1 β-strands responsible for catch bond formation are instead involved in the strand-swapping. Finally, the lifetime of a structural intermediate, corresponding to the transition from the X-dimer to strand-swapped configuration, is independent of the applied force. This mechano-insensitivity arises due to a torsional rotation generated perpendicular to the applied pulling force causing protein unbinding that does not depend on the magnitude of the force.

The intracellular domain of cadherin is linked to β-catenin which binds, via α-catenin, to the actin network^124^. A reconstruction of the cadherin-catenin-actin complex in solution shows that while the cadherin and catenin forms a stable connection, the interaction formed with actin is surprisingly weak^125^, at odds with a stable connection observed in cells ^126,127^. This dichotomy was resolved upon the application of force to a cadherin-catenin-actin complex that mimics the adherens junction geometry^128^. In this configuration, the binding dynamics of the cadherin-catenin complex with actin exhibits catch-bond behaviour. Further characterization reveals that the interaction strength between β-catenin/α-catenin is stable, with survival times ranging from 10-100s under the application of physiologically relevant forces up to 10 pN^129^. However, the binding lifetime under force is reduced upon the introduction of two physiologically relevant phosphorylation sites known to disrupt tight junctions (tyrosine 142 or threonine 120).

Single molecule experiments performed on isolated catenins reveal that the mechanical unfolding of β-catenin is triggered at low physiological forces, but the unfolding pathway is highly heterogeneous, exhibiting large variability in the force and contour length associated to a single protein unfolding event^130^. On the other hand, SmMT experiments performed on a truncated construct of the aE isoform and the aT(testes) isoform of catenin revealed that α-catenin undergoes three steps when mechanically unfolding, the first one occurring at a low force (∼5 pN) followed by two consecutive step-wise events requiring higher forces (∼7-15 pN) to unfold^131,132^. This is a fully reversible process, whereby each region of the protein can successfully refold into its native structure in the absence of force. Interestingly, the mechanically vulnerable region of α-catenin contains a cryptic vinculin binding site that is inaccessible when the protein is in its native folded conformation. Analogous stretching experiments performed in the presence of purified vinculin reveal that vinculin binding occurs after mechanical unfolding^131^. Noteworthy, upon vinculin binding, α-catenin is locked into an unfolded and flexible state that does not fold in the absence of force. The bound vinculin is only expelled from α-catenin during a high force pulse, which presumably sufficiently alters the structural configuration of the binding site such that binding is no longer sustained. These experiments are reminiscent of the behaviour observed in talin at the focal adhesion sites^96^, suggesting that cells may have a handful of conserved molecular mechanisms that are replicated in different regions of the cell to regulate the connection and communication with the external environment.

Altogether, this work offers mechanistic insight into how molecular tethers at the cell-cell interface in adherens junctions utilizes mechanical force to tune the interaction lifetime of proteins, individual protein conformation and reactivity to regulate cellular attachment and signalling. Our current understanding arising from force spectroscopy experiments is mainly focused on the characterization of proteins located at adherens junctions. However, to build up a comprehensive picture of how mechanical force governs cell-cell adhesion, this work must extend to cover the proteins found in other cell attachment complexes such as tight junctions, and desmosomes.

## Single molecule mechanosensing in the cytoskeleton

**Figure 3:**
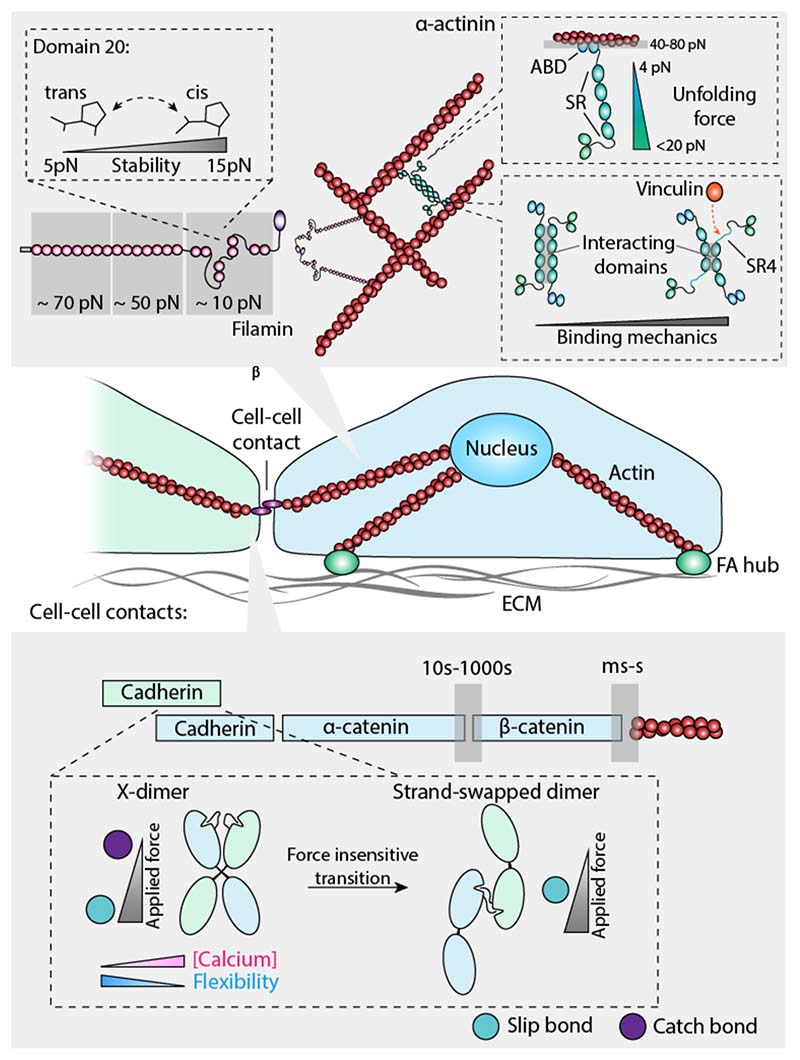
Mechanical force regulates the nature of interactions within the cytoskeleton and between cells. (*Upper panel*) Single molecule force spectroscopy experiments reveal the plethora of mechanisms employed by actin binding proteins to regulate their mechanical properties. FilaminA exhibits hierarchy in the mechanical stability, ranging from 10-70 pN. The mechanical properties of the domain 20 switches between 5 pN and 20 pN depending on the conformation of a single proline. Furthermore, the α-actinin protein, which forms a mechanically stable connection with actin, regulates vinculin binding depending on the conformation of the 4^th^ domain of the spectrin-like region. (*Lower panel*) The cadherin-catenin complex maintains the mechanical connection spanning from the extracellular interface to the actin cytoskeleton. The conformation of the cadherin dimer and the calcium concentration dictate the mechanical response of the interaction. The X-dimer exhibits both catch- and slip-bond behaviour depending on the magnitude of applied force. By contrast, the strand-swapped dimer only exhibits slip-bond behaviour.

The actin cytoskeleton – which links focal adhesions at the plasma membrane to the Linker of Cytoskeleton (LINC complex)^133^ at the nucleus — is a dynamic network that responds to a variety of mechanical stimuli via polymerization/depolymerization. Furthermore, adapter proteins bind to the actin cytoskeleton and respond to forces by regulating their elasticity through mechanical (un)folding. Consequently, actin-binding proteins (ABPs) regulate actin polymerization indirectly by tuning their elasticity (Figure 3).

Filamins are ABPs that link neighbouring fibres and create an aligned network, and also function as signalling hubs by recruiting proteins to the network, or anchoring actin to transmembrane receptors^134^. This diverse functionality is afforded by a few structural elements. Firstly, filamins exist as homodimers, linked at the C-terminal. At the N-terminal there is an actin binding domain, such that each homodimer spans two actin fibres. The termini are separated by a region of Ig domains, ranging from 6-24 depending on the filamin isoform. Filamin A, the most studied with force spectroscopy, contains 24 Ig domains^135^. Domains Ig1-15 adopt a linear conformation, and exhibit a range of unfolding forces spanning 50-200 pN^136–138^. Loading rates closer to the dynamics of actin (1.6pN/s)^139^, reveal a mechanical hierarchy along the linear Ig-domains, with higher stability for Ig1-8 (∼70 pN) and lower towards for Ig9-15 (∼ 50pN). Domains Ig16-23 assemble into a compact structure, arranged in domain pairs such that the A-strand of the even numbered domains binds to the subsequent odd numbers, thus creating a binding interface between the neighbouring domains. These domains all unfold at forces ∼10 pN, which is compatible with the forces produced by a few myosin motors acting on actin^140^. The low mechanical stability is attributed to the domain-pair configuration, which gives rise to a different pulling geometry causing an unzipping of the interface^139^.

Closer examination of Ig20 reveals a dual mechanical stability, interchangeably switching between a low unfolding force (∼5 pN) and a higher force (∼15 pN)^141^. This mechanical switch is attributed to the isomerization of a highly conserved proline close to the last β-strand of the domain, which can exist in a cis- or trans-conformation^142^. The addition of the peptidyl−prolyl isomerase enzyme SlyD accelerates the trans-to-cis conversion^143^. Mechanically stretching the Ig20-21 of filamin A in the presence of three known binding partners (namely, the cytoplasmic tail of integrin β7, a fragment of glycoprotein Ib and a fragment of the integrin regulator migfilin) reveals that the binding affinity between the domain pair and binding ligand is highly dependent on the magnitude of mechanical force, whereby a shift from 2 pN to 5 pN is sufficient to change the binding affinity by a factor of 17^144^.

The ABP α-actinin plays an important role in actin organization by cross-linking nearby actin fibres^145^. α-actinin is composed of a mechanically stable N-terminal actin binding domain (*F*>20 pN), four central spectrin-like rod domains (∼18-27 pN) and finally a weak C-terminal calmodulin-like domain composed of four calcium binding EF hands (∼4-10 pN)^146^. The actin and α-actinin connection is mechanically robust, requiring forces ∼40-80 pN (at pulling velocities between 4-50pN/s) to rupture. Furthemore, α-actinins self-assemble into anti-parallel dimers with the actin-binding domain at either end. This dimer binding interface readily ruptures (∼5 pN), and rebinds (∼0.6 pN) according to the magnitude of applied force. An alternate partially-connected dimer conformation, where the two central spectrin-like domains are connected and the two outer domains are disconnected, permits the mechanically weak SR4 domain to unfold, thus lowering the tension to sustain the dimer interaction. Unfolding of the SR4 domain initiates vinculin binding, which then locks the SR4 domain in an unfolded conformation. Protein refolding only occurs when the SR4 domain is triggered to undergo a helix-to-coil transition at high (∼35 pN) pulling forces which triggers the expulsion of the bound vinculin.

The ABP Gelsolin organizes the network by severing, capping and uncapping the actin fibres, a process tightly regulated by the concentration of calcium ions^147^. In particular, the ^6th^ domain of gelsolin contains a single calcium binding site, which, when occupied, undergoes a conformational change^148^. Single molecule experiments reveal that the gelsolin’s mechanical stability gradually increases from ∼24 pN to 41 pN upon the addition of 50 uM calcium^149^. Experiments in conjunction with a force-dependent kinetic model reveal that mechanical forces across the 6^th^ domain of gelsolin modulate the binding affinity for Ca^2+^ by decreasing the dissociation constant, thus giving a more stable calcium-bound state under force. This may serve as a mechanism within the actin network to recruit calcium ions to regions of high tension, leading to highly localised changes in signalling in response to force.

Formins function as homodimers via an FH2 domain, assembling into ring-like structures that encircle the barbed end of actin filaments, and regulate the polymerization process, generally resulting in a slower polymerization^150,151^. The N-terminal of the FH2 domain is linked to an intrinsically disordered FH1 domain composed of multiple polyproline tracks, which has a high binding affinity to the ABP proflin, generating an enrichment of proflin at the barbed end of actin which facilitates polymerization^152^. Pulling forces applied to actin (from as low as 0.5 pN) in the presence of a freely rotating formin protein (mDia1) significantly increase the actin elongation rate^153,154^. If rotational movement is restricted, the polymerization rate is significantly slower^154^. Additional experiments dissecting the mechanosensitive components of mDia1 revealed that the FH2 domain, and not the FH1 domain, is the region of the protein predominantly responsible for the force-sensing capability of the protein^155^. Mechanistically, mechanical force drives a conformational change in mDia1, switching from a closed and inhibitory ring to an open and active ring that drives polymerization.

These experiments have uncovered an arsenal of approaches utilized by the cell to regulate cytoskeletal organization in response to mechanical stimuli. However, given the complexity of the cell cytoskeleton, and the breadth of proteins involved, we are still far from a comprehensive understanding of the mechanisms governing cytoskeleton mechanics and mechano-regulation. Furthermore, our current understanding of cellular mechanics is very ‘actin-centric’, with the majority of single molecule experiments focusing on actin-binding proteins and largely overlooking the potentially important role of intermediate filaments and microtubules.

## Nanomechanical regulation of muscle contractility by the sarcomeric cytoskeleton

A very specialised case of cytoskeletal structure can be found in the muscle sarcomere^156^. Due to the highly organised structure of muscle (based on the regular assembly of sarcomeres^157^, which are, in turn, organised in an array of precisely connected polypeptides), it is not surprising that muscle contractility has provided an excellent biological system to directly probe the effect of force on the conformational dynamics of the key proteins forming the unique sarcomeric cytoskeleton, underpinning mechanical function. The muscle mechanics field typically distinguishes between the ‘active’ forces^158^ – dependent on the action potential and subsequent Ca^2+^ release – provided by the actomyosin contractility and the ‘passive’ forces generated by titin filaments, whose function is directly determined by protein elasticity^159^.

**Figure 4:**
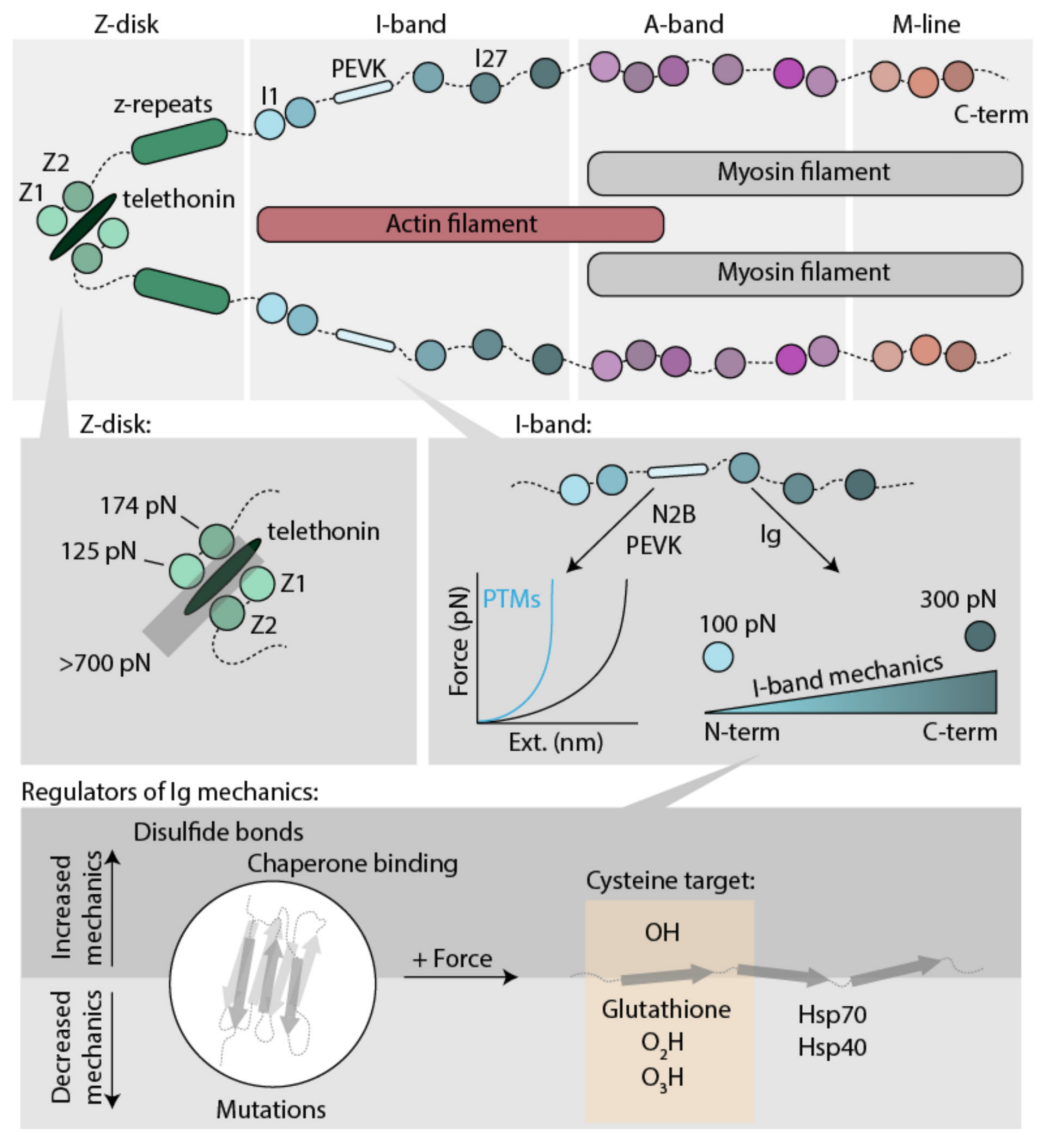
Lessons of muscle nanomechanics learnt with single molecule techniques. The giant protein titin is responsible for the passive elasticity of muscle, and has arguably been the most studied protein at the nanomechanical level. (Upper panel) From a molecular perspective, titin is formed by a series of mechanically stiff Ig domains that work (that work as shock absorbers) intercalated by intrinsically disordered sequences (that behave like entropic springs). (Lower panel) Several molecular strategies, encompassing chaperone and ligand binding and a wide range of post-translational modifications, have been shown to affect titin nanomechanics.

The giant titin protein is alternatively spliced and connects the Z- and M-lines of the striated muscle sarcomeres, guaranteeing their mechanical and structural integrity^160^ (Figure 4). The NH_2_-terminal titin segment is firmly anchored to the Z-disk through alpha-actinin and actin^161^, ensuring titin’s role as a molecular ruler for sarcomere assembly. This firm attachment involves the Z1 and Z2 domains (which independently unfold at 125 pN and 174 pN)^162^ of two parallel titin molecules interacting in a palindromic way, mediated by telethonin (T-cap), which acts as a molecular glue that forms a ultrastable (∼707 pN) molecular bond^163^. Surprisingly, even in the absence of telethonin, the forces required to mechanically disrupt titin Z1Z2 dimers also approach ∼700 pN^162^. In the I-band, there is an overall relationship between Ig position and their mechanical stability; domains from the proximal region show a relatively low mechanical stability [e.g. Ig1 (130 pN)^16^4, Ig4 (171 pN) and Ig5 (55 pN)^165^, Ig10 (130pN)^166^], whereas Ig domains from the distal region exhibit a strong mechanical phenotype, which generally increases along the Z-line to M-line direction [eg. Ig 27 (204pN), Ig28 (257pN), Ig32 (298pN) and Ig34 (281pN)^165^. Crucially, unstructured regions (N2B and PEVK) that behave like perfect entropic springs^167,168^ (although low force MT measurements revealed an enthalpic yet labile structure in N2B^169^) are intercalated between the stiffer Ig shock absorbers. Therefore, when force is applied, the linkers between the different domains are straighten out; then the unstructured PEVK and N2B regions entropically extend, followed by the unfolding of the mechanically labile Ig domains of the proximal region, being the mechanically stiffer Ig domains of the distal region the last to unfold. Hence, the ‘elastic’ I-band, which is alternatively spliced, determines the overall titin’s spring constant. By contrast, the A-band and M-band regions, which are thought to be mechanically rigid and inextensible, determine the actomyosin interaction zone.

Whether mechanical unfolding of the Ig domains occurs in vivo has been—and continues to be— a matter of intense debate. The high mechanical stability of the Ig domains have been long considered non-physiological. However, recent SmMT observations revealed that several Ig domains from the proximal region unfold and refold in equilibrium at physiological forces (e. 4-8pN)^170^. Furthermore, isolated myofibers labeled with quantum dots stretched to 3 µm sarcomere length (considered to be within the physiological range), display stepwise changes in the distance between individual pairs of Qdots, reminiscent of individual unfolding and refolding events. In fact, the ability of individual titin domains to refold against a pulling force, therefore generating mechanical work, has been put forward as a previously unappreciated mechanism, complementary to the active actomyosin contractility, of energy generation in the contracting muscle^171^. This enticing yet controversial hypothesis will need further experiments to be dis/proved. Similarly, the unambiguous demonstration that titin’s Ig unfolding and refolding under force as a physiological mechanism that muscle exploits to regulate extensibility warrants definitive in-vivo experiments. Two independent recent SmMT experiments reported an unexpected softening of the Ig27 domain at 37°C^172^, and an increase in its unfolding rate at low pulling forces^173^, suggesting that the forces required to unfold titin’s Ig domains under physiological conditions might be indeed lower than previously anticipated.

Combined, these experiments demonstrate that titin acts as a molecular ruler controlling muscle elasticity by exploiting the mechanical hierarchy of its building blocks to control extensibility. However, titin elasticity is not solely controlled by its molecular architecture; other protein-specific mechanisms including force-induced post-translational modifications and ligand binding affect titin’s response to force. When exposed to glutathione, previously cryptic cysteines form a mixed disulfide bond results in a mechanically weaker Ig domain with impaired refolding capabilities^174^. Similarly, S-sulfenylation leads to an oxidized protein devoid of mechanical stability, unless it condenses with a close-by cysteine to create a rigid disulfide bond that endows the protein with increased stiffness^175^. In general, the interplay between disulfide formation and S-thiolation underscores a general mechanism to dynamically regulate titin stiffness through chemical reactivity^60^, especially since many I-band Ig domains contain at least 3 evolutionary conserved cysteines. This, in turn, enables SS isomerization as a potential master regulator of protein elasticity^176^. Similarly, under oxidizing conditions, the unstructured N2B sequence exhibits a much shorter extensibility when compared to reduced conditions, compatible with the presence of newly formed non-native disulfide bonds, which render the overall protein stiffer^177^.

In addition, phosphorylation in the compliant PEVK and N2B regions by protein Kinase C significantly decreases its persistence length, hence increasing the cardiomyocyte passive tension^178^. By contrast, the persistence length of the N2B segment increased upon treatment with cGMP-activated kinases, such as PKG, ERK2 and CaMKIIδ,^179,180^ resulting in a decrease in the passive cardiomyocyte tension. Protein binding also offers an alternative general mechanism to modulate elasticity. In particular, the αB-crystallin chaperone slightly increases the mechanical stability of Ig domains^181^. Moreover, both Hsp40 (DnaJ) and DnaK (Hsp70) chaperones work as a ‘holdase’ by binding to the collapsed states of Ig27, hindering refolding and resulting in a mechanically weak polypeptide. By contrast, in the presence of ATP, the whole DnaJKE system significantly promotes refolding of the slow-folder Z1 Ig domains^182^. These results reveal chaperone binding as an effective regulatory mechanism of protein elasticity, although the binding of chaperones to a mechanically stretched protein is sequence-specific. Single molecule experiments have also been key to unravel the molecular mechanisms underpinning the general role of titin as a mechanoactivated signalling hub^183^. For example, mechanical force can trigger ATP binding to the M-band titin kinase domain^184^, involved in muscle gene transcription and load-dependent protein turnover, by exposing its previously cryptic site by mechanically unfolding its autoinhibitory domain as predicted by SMD simulations^185^.

While titin is the main (and largest) molecular spring in the muscle, a diverse suite of proteins need to guarantee titin’s firm anchoring to both thick filaments through the A-band and M-line on one end, and, to the Z-disk at the other end, fulfilling both structural and signalling roles^183,186^. In particular, α-actinin anchors titin to the Z-disk. Curiously, the titin/α-actinin interaction withstands very low forces (1-3.5 pN). However, the force-dependency of the interaction life-time led to a model based on a cooperative effect of multiple titin/α-actinin engaged at a given time to explain the long-term stability of the interaction^187^. In the M-band, a ternary complex between titin, obscurin (or obscurin-like-1) and myomesin is responsible for its structural scaffold function as a mechanical hub. In particular, the binding interaction between the titin M10 domain and obscurin is relatively weak (∼30 pN)^188^. This suggests that even moderate weakening of the interaction might be of physiological relevance, and that the concerted binding of the protein network might be crucial to preserve the mechanical integrity of the M-band. Nanomechanical experiments on the myomesin dimer revealed the molecular origin of its elasticity, encompassing the reversible stretching of the α-helix linkers between the My12 and My13 Ig domains (∼40 pN)^189^ and in general between the My9-My13 Ig domains^190^, followed by the mechanical unfolding of the Ig domains and the final dissociation of the dimerization interface (∼137 pN). In that vein, the physical connection between obscurin(-like-1) and myomesin was found to involve a trans-complementary mechanism between the My4 and My5 Ig domains and the 3^rd^ Ig domain of obscurin(-like-1). Such a swapped conformation (due to myomesin complementation) provided mechanical protection to the mechanically more labile obscurin O3 domain^191^.

Combined, this rich body of literature provides a first molecular glance on the mechanisms underpinning the resting elasticity of muscle. However, given the number of involved proteins, the complexity of the binding networks and the intrinsically distinct dynamics under force of each individual molecular player, there is probably a wealth of still elusive knowledge to fully understand nanomechanical architecture of muscle elasticity. The mechanical behaviour of a number of unstudied molecular partners, the dynamic modifications of protein elasticity through PTMs or ligand binding, or the possible energetic coupling between the ‘active’ actomyosin machinery and the – thus far considered – passive titin-mediated elasticity will need to be experimentally addressed in the near future.

## Nuclear proteins

Compared to the large number of proteins investigated within the context of focal adhesion and cytoskeletal links, the nanomechanical properties of nuclear proteins have been comparatively much less studied. In fact, the only two examples are the different regions of nuclear lamins, which are type V intermediate filaments that form an elastic meshwork underlying the nuclear membrane. While the 1B and 2B domains of the lamin rod exhibit varying extensions and several mechanical intermediates due to their coiled-coil structure^192^, the Ig domain of lamin A (and its pathogenic mutants) unfolds in a two-state manner, albeit at relatively low forces (∼50pN)^193^, hence close to the limit for AFM detection.

## Novel mechanical functions in biology unravelled by single molecule mechanics

The majority of the work reviewed above refers to proteins with a relatively obvious mechanical role in vivo. However, recent single molecule experiments have uncovered a number of key biological processes —namely those related to the genesis and degradation of proteins— where mechanical force, perhaps surprisingly, also plays a fundamental role.

For example, the folding of a nascent polypeptide that emerges from the exit tunnel of the ribosome exerts a pulling force that can rescue SecM-stalled ribosomes^194^, suggesting that a protein can regulate its own synthesis by the force generated during co-translational folding.

At the other end of the duty cycle of (bacterial) proteins, proteins that translocate across the narrow pore of the ClpX/ClpP proteolytic machinery must first mechanically unfold^195–197^. Crucially, the protein substrates chosen for these proof-of-principle experiments mostly involved model proteins the mechanical stability of which had been previously assessed with single molecule experiments, such as filamin domains^137^, the I27 Ig domain of titin^198^, the halo protein^199^ or GFP^200^. Using titin I27 mutants previously characterised^54^, it was shown that the mechanically less stable I27_V15P_ and I27_V13P_ mutants required less time to unfold than the mechanically resistant I27_WT_, suggesting that the mechanical stability of each protein determines its unfolding rate. Further experiments showed that the unfolding rate (but not so much the translocation rate) of I27_WT_ and I27_V15P_ by ClpX/ClpP is higher when the proteins are pulled from the N-terminus, concluding that mechanical degradation of proteins is highly dependent on the pulling direction^201^. Combined, these experiments demonstrated the power of single molecule technologies to unravel the physical principles underpinning a variety of novel force-activated biological processes that are, directly or indirectly, regulated by the conformational dynamics of proteins under force.

## Minding the gap: from single molecule to single cells

Single molecule force spectroscopy experiments have revealed a sophisticated repertoire of molecular strategies to regulate protein mechanical stability. However, the focus is shifting towards elucidating whether the behaviour of individual proteins under force can be translated to the cellular scale. Particularly compelling questions include to which extent proteins mechanically unfold in vivo, and whether the principles determining in vitro behaviour can be applied to predict behaviour at the cellular scale. While evidence of mechanical force regulating the conformational dynamics of proteins in their native cellular environment has emerged through the years, a handful of recent studies showing a direct connection of the mechanical behaviour of proteins across length scales have been reported.

Early biochemistry-supported cellular experiments provided evidence that proteins mechanically unfold in vivo. For example, combining cryptic cysteine labelling with quantitative mass spectrometry showed that hidden cysteines of in a range of force-bearing proteins^202^ became increasingly labelled when erythrocytes were exposed to shear stress. The sensitivity of the methodology enables identification of the precise protein region that becomes exposed upon forced unfolding^203^. For example, the partially buried Cys522 in the Ig domain of laminA becomes progressively labelled when isolated nuclei are exposed to higher shear stress^4,204^.

**Figure 5:**
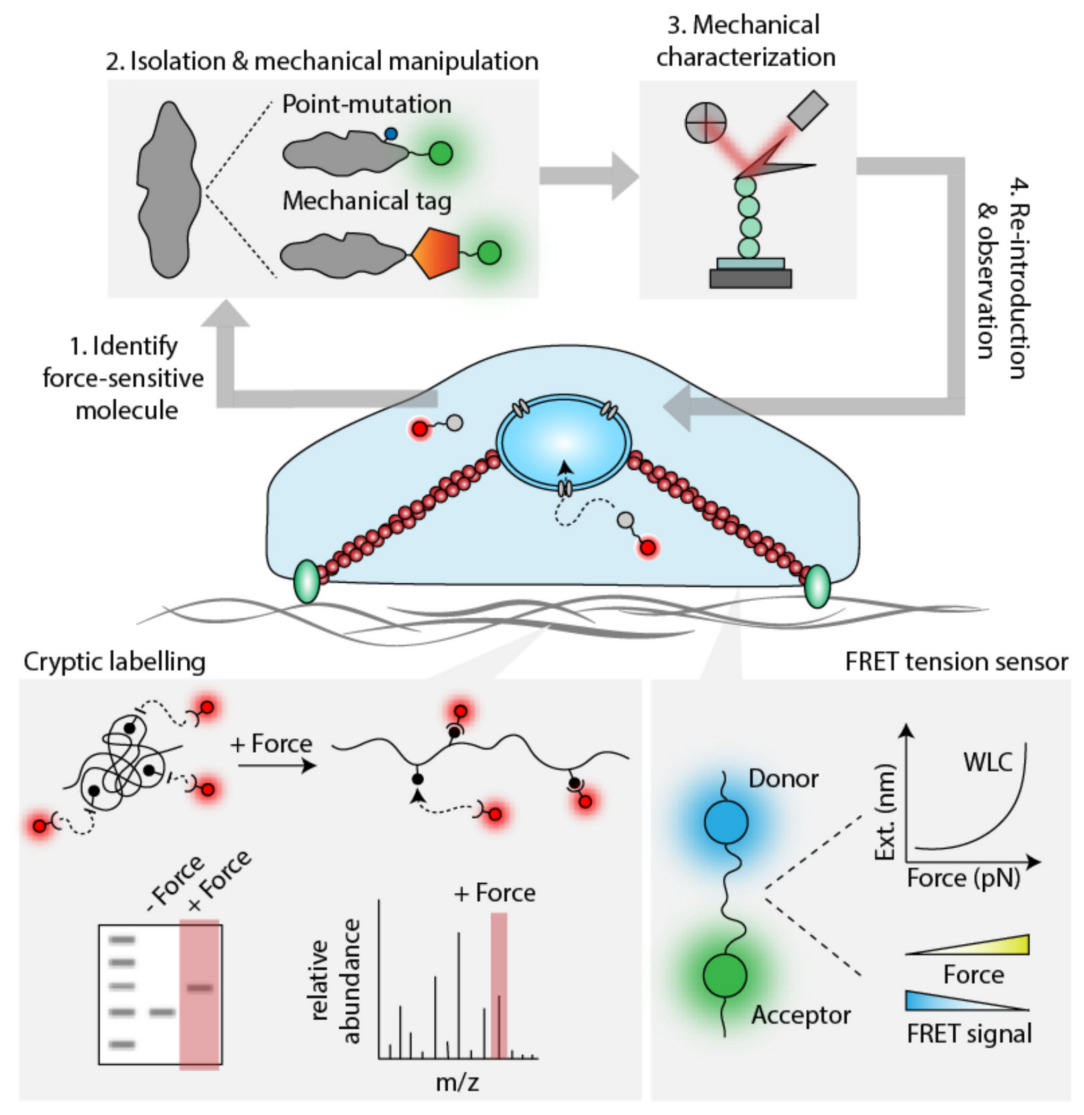
Integrating single molecule experiments into cells. A number of emerging strategies aim to translate our single molecule understanding into the cellular context. A first general and promising approach modifies a single molecule either by the introduction of a point mutation or by the addition of a mechanical tag. These constructs can then be extensively characterized using single molecule approaches and then introduced into the cell. Subsequent changes in the cellular behaviour can then be directly attributed to changes in the mechanical properties of the individually modified protein. Alternatively, upon understanding the conformational changes undergone upon mechanical unfolding, it is possible to design probes that will specifically target cryptic protein residues that are exposed upon unfolding. The probe binding can be detected via western blots, mass spectrometry or fluorescence microscope. Therefore, these probes function as a direct readout of mechanical unfolding. Finally, FRET tension sensors combine the concepts of fluorescence resonance transfer with polymer physics. In this configuration, a donor and an acceptor fluorophore are separated by a mechanically characterized linker such that the FRET signal is a readout of the mechanical force applied to the system

The force-exposure of previously buried residues can result in post-translational modifications which facilitate new binding interactions and initiate downstream mechanosignalling^205^. For example, mechanical force applied to focal adhesions unfolds p130Cas, revealing cryptic tyrosine residues that are susceptible to phosphorylation by Src kinases^206^. Similarly, mechanically stretching isolated nuclei triggers emerin phosphorylation at Tyr-74 and Tyr-95 by Src kinase^207^ leading to nuclear stiffening. Conversely, mechanical stress induces phosphorylation of the nuclear envelope lamins (A/C and B type) leading to nuclear softening^4^. Taken together, these changes in cellular elasticity require a thorough understanding of the mechanical deformation of the molecular players. Yet a direct connection between the force-induced conformational changes in single proteins and the knock-on effects at the cellular level remain restricted to a handful of exciting reports (Figure 5).

For example, to understand the first stages of cellular mechanotransduction in focal adhesions, the mechanically stable IVVI talin mutant characterised by SmMT^96^ to show that nuclear translocation of the YAP transcription factor requires talin unfolding^208^. In this vein, cells expressing a disulfide mutant of talin’s R8 domain with impaired extensibility (characterised at the single molecule level) lock talin in the DLC binding-active native state, resulting in altered focal adhesion dynamics, lower actomyosin contractility and impaired cell migration^103^.

At the junction level, Spadaro et al. demonstrated that in the epithelial tight junctions, the conformation of the ZO-1 protein depends on actomyosin-generated forces, which leads to modified gene expression and epithelial morphogenesis^209^. SmMT experiments revealed the forces (2-4 pN) required to keep the full-length ZO-1 in a stretched yet still folded conformation. These experiments provided a first molecular glimpse on the force-induced conformational changes regulating the dynamics of the epithelial junctional proteins.

In the context of muscle, Alegre Cebollada et al demonstrated, also spanning from single molecules of titin Ig domains to skinned cardiomyocytes, that glutathionylation of cryptic cysteines in titin prevents protein refolding, converting the Ig domains from shock absorber into elastic springs^174^. Expanding the length-scale gap further, Rivas-Pardo et al severed a HaloTag-TEV insertion in the titin protein of a mouse model to quantify the passive force generated by titin in skin myocytes and then performed a nanomechanical study of the severed proteins using SmMT^210^.

Recently, nanomechanical experiments have uncovered mechanical aspects defining nuclear transport^211,212^. By attaching proteins of varying mechanical stabilities characterised at the single molecule level (ranging from ∼15 to 700 pN)^54,59,165,213^ to particular transcription factors, such as YAP^212^ or MRTF-A^211^, or to nuclear optogenetic tools, it was demonstrated that the mechanical — and not the thermal —stability of proteins regulates their translocation rate to the nucleus, with direct effects on gene expression and cell function.

Collectively, these experiments, involving proteins from different locations across the cell, demonstrate that the nanomechanical experiments conducted in vitro are a useful proxy to begin to understand the mechanical behaviour of individual proteins within their physiological cellular context. However, and despite these meritorious efforts aiming at closing the length-scale gap, those cross-scale experiments are still far from definitive, and many standing questions remain. First and foremost, we do not know the absolute values of the forces experienced by proteins inside the cell and to which extent the forces measured in vitro using single molecule force spectroscopy can be translated when working in the cellular environment.

To begin to address this question, molecular tensors have provided a *tour-de-force* towards the measurement of intracellular molecular forces^214,215^. The general approach consists of an elastic peptide flanked by two fluorophores. The mechanical stability of the peptide sets the measurable force range. Since Förster resonance energy transfer (FRET) is distance-dependent, as the mechanosensitive peptide extends under force, the FRET signal is reduced. For example, a tension sensor placed within different regions of the vinculin structure demonstrated that the tension across vinculin in stable focal adhesions is 2.5 pN^216^. A higher-force biosensor revealed that talin is exposed to forces >7 pN during cell adhesion, and uncovered that the range of forces depends on the degree of its mechanical engagement with vinculin and actin^217^. A follow-up molecular design of a new biosensor exhibiting a narrow (3-5 pN) force sensitivity range that is also capable of multiplexing different force sensors within the same cell demonstrated an intramolecular tension gradient across talin-1 that follows integrin-mediated adhesion^218^. While most of these genetically-encoded FRET molecular sensors have explored the forces borne by individual proteins at the FA or Junction levels, inserting a sensor within nesprin-2G, a key component of the LINC complex, enabled direct force measurement at the nuclear envelope level^219^. Despite these tremendous advancements, the limitations of the genetically-encoded tensors are still significant. For example, there is an intrinsic limitation in the upper limit of forces measurable with currently available biosensors set at about 11 pN, hence leaving a wide range of potentially higher forces experimentally unmeasurable. Moreover, forces can only be measured across sensor-tagged proteins, and the resulting measurements do not provide information on the resultant orientation of the force vector. More importantly, it is unknown to which extent the in-vitro experiments provide an unambiguous calibration of the sensor when genetically encoded inside the cell; in particular, we do not know the velocity at which proteins are being pulled inside the cell, and whether effects such as crowding might affect the effective mechanical stability of the stretched proteins in the cellular context.

## Concluding remarks

The single molecule nanomechanics field, spurred by continuous technological improvements, has shown that protein structure and chemical reactivity need to combine with concepts of polymer physics to provide a comprehensive understanding of the dynamics of single proteins under force. In particular, mechanical force can tune protein binding at two distinct levels; first, mechanical force unfolds proteins and exposes previously cryptic binding pockets. Secondly, and once unfolded, mechanical force may regulate binding/unbinding kinetics. Alternatively, binding can occur in the protein’s native state, potentially affecting the protein’s mechanical stability.

Additionally, a variety of force-induced post-translational modifications affect protein mechanical stability, and their subsequent unfolding/refolding dynamics. However, we do not know whether exposed amino acids have a similar probability of undergoing post-translational modifications, and, whether all post-translational modifications will impact the protein’s mechanical properties.

Arguably the main standing challenge is to establish a direct connection with physiology in a quantitative manner. The first step involves connecting the behaviour of proteins measured in vitro with the cellular environment. This is challenging given that the cellular environment is crowded and viscous. We don’t know how proteins will mechanically un/refold under these conditions. Or whether the sensitivity to force will remain unaltered as proteins sample physiological regimes of ‘pulling velocities/stretching forces’. What are ‘physiological’ forces is also a matter of debate. On the one hand, strong external mechanical impacts might need to dissipate large amounts of energy over short timescales. On the other hand, homeostatic functioning of the cells and tissues might require much lower forces. Answering these questions involves a titanic mission of measuring forces within individual proteins inside cells. We are currently far from this, yet the first cross-scale experiments are extremely encouraging. Only then will we be able to start asking more complex questions, such as how many individual proteins withstand a given mechanical perturbation, how synchronised and directional is mechanical unfolding/refolding within the cell, or how much individual mutations (or posttranslational modifications, or binding events) are required to elicit explicit functional effects at the cellular and tissue levels and beyond.

## References

[1] Iskratsch T, Wolfenson H, Sheetz MP (2014). Appreciating force and shape-the rise of mechanotransduction in cell biology. Nat Rev Mol Cell Biol.

[2] Dumortier JG (2019). Hydraulic fracturing and active coarsening position the lumen of the mouse blastocyst. Science.

[3] Engler AJ, Sen S, Sweeney HL, Discher DE (2006). Matrix elasticity directs stem cell lineage specification. Cell.

[4] Swift J, Discher DE (2014). The nuclear lamina is mechano-responsive to ECM elasticity in mature tissue. J Cell Sci.

[5] Vining KH, Mooney DJ (2017). Mechanical forces direct stem cell behaviour in development and regeneration. Nat Rev Mol Cell Biol.

[6] Wang N, Tytell JD, Ingber DE (2009). Mechanotransduction at a distance: mechanically coupling the extracellular matrix with the nucleus. Nat Rev Mol Cell Biol.

[7] Tajik A (2016). Transcription upregulation via force-induced direct stretching of chromatin. Nat Mater.

[8] Kirby TJ, Lammerding J (2018). Emerging views of the nucleus as a cellular mechanosensor. Nat Cell Biol.

[9] Nava MM (2020). Heterochromatin-Driven Nuclear Softening Protects the Genome against Mechanical Stress-Induced Damage. Cell.

[10] Huse M (2017). Mechanical forces in the immune system. Nat Rev Immunol.

[11] Jaalouk DE, Lammerding J (2009). Mechanotransduction gone awry. Nat Rev Mol Cell Biol.

[12] Paul CD, Mistriotis P, Konstantopoulos K (2017). Cancer cell motility: lessons from migration in confined spaces. Nat Rev Cancer.

[13] Mohammadi H, Sahai E (2018). Mechanisms and impact of altered tumour mechanics. Nat Cell Biol.

[14] Dufrene YF, Persat A (2020). Mechanomicrobiology: how bacteria sense and respond to forces. Nat Rev Microbiol.

[15] Roca-Cusachs P, Conte V, Trepat X (2017). Quantifying forces in cell biology. Nat Cell Biol.

[16] Campas O (2014). Quantifying cell-generated mechanical forces within living embryonic tissues. Nat Methods.

[17] Hu X, Margadant FM, Yao M, Sheetz MP (2017). Molecular stretching modulates mechanosensing pathways. Protein Sci.

[18] Stirnemann G, Giganti D, Fernandez JM, Berne BJ (2013). Elasticity, structure, and relaxation of extended proteins under force. Proc Natl Acad Sci U S A.

[19] Cecconi C, Shank EA, Bustamante C, Marqusee S (2005). Direct observation of the three-state folding of a single protein molecule. Science.

[20] Shank EA, Cecconi C, Dill JW, Marqusee S, Bustamante C (2010). The folding cooperativity of a protein is controlled by its chain topology. Nature.

[21] Fernandez JM, Li H (2004). Force-clamp spectroscopy monitors the folding trajectory of a single protein. Science.

[22] Neupane K (2016). Direct observation of transition paths during the folding of proteins and nucleic acids. Science.

[23] Yu H, Siewny MG, Edwards DT, Sanders AW, Perkins TT (2017). Hidden dynamics in the unfolding of individual bacteriorhodopsin proteins. Science.

[24] Choi HK (2019). Watching helical membrane proteins fold reveals a common N-to-C-terminal folding pathway. Science.

[25] Petrosyan R, Narayan A, Woodside MT (2021). Single-Molecule Force Spectroscopy of Protein Folding. J Mol Biol.

[26] Bustamante C, Alexander L, Maciuba K, Kaiser CM (2020). Single-Molecule Studies of Protein Folding with Optical Tweezers. Annu Rev Biochem.

[27] Schonfelder J, Alonso-Caballero A, De Sancho D, Perez-Jimenez R (2018). The life of proteins under mechanical force. Chem Soc Rev.

[28] Sharma S, Subramani S, Popa I (2021). Does protein unfolding play a functional role in vivo?. FEBS J.

[29] Garcia-Manyes S, Beedle AEM (2017). Steering chemical reactions with force. Nature Reviews Chemistry.

[30] Veigel C, Schmidt CF (2011). Moving into the cell: single-molecule studies of molecular motors in complex environments. Nat Rev Mol Cell Biol.

[31] Krieg M (2018). Atomic force microscopy-based mechanobiology. Nature Reviews Physics.

[32] Neuman KC, Nagy A (2008). Single-molecule force spectroscopy: optical tweezers, magnetic tweezers and atomic force microscopy. Nat Methods.

[33] Moffitt JR, Chemla YR, Smith SB, Bustamante C (2008). Recent advances in optical tweezers. Annu Rev Biochem.

[34] Yang B, Liu Z, Liu H, Nash MA (2020). Next Generation Methods for Single-Molecule Force Spectroscopy on Polyproteins and Receptor-Ligand Complexes. Front Mol Biosci.

[35] Milles LF, Schulten K, Gaub HE, Bernardi RC (2018). Molecular mechanism of extreme mechanostability in a pathogen adhesin. Science.

[36] Milles LF, Gaub HE (2020). Extreme mechanical stability in protein complexes. Curr Opin Struct Biol.

[37] Schoeler C (2014). Ultrastable cellulosome-adhesion complex tightens under load. Nat Commun.

[38] Liu Z (2020). High force catch bond mechanism of bacterial adhesion in the human gut. Nat Commun.

[39] Herman-Bausier P, Dufrene YF (2018). Force matters in hospital-acquired infections. Science.

[40] Mathelie-Guinlet M (2020). Force-clamp spectroscopy identifies a catch bond mechanism in a Gram-positive pathogen. Nat Commun.

[41] Viela F, Speziale P, Pietrocola G, Dufrene YF (2019). Mechanostability of the Fibrinogen Bridge between Staphylococcal Surface Protein ClfA and Endothelial Cell Integrin alphaVbeta3. Nano Lett.

[42] Bustamante CJ, Chemla YR, Liu S, Wang MD (2021). Optical tweezers in single-molecule biophysics. Nature Reviews Methods Primers.

[43] Edwards DT (2015). Optimizing 1-mus-Resolution Single-Molecule Force Spectroscopy on a Commercial Atomic Force Microscope. Nano Lett.

[44] Cecconi C, Shank EA, Marqusee S, Bustamante C (2011). DNA molecular handles for single-molecule protein-folding studies by optical tweezers. Methods Mol Biol.

[45] Mora M, Stannard A, Garcia-Manyes S (2020). The nanomechanics of individual proteins. Chem Soc Rev.

[46] Lof A (2019). Multiplexed protein force spectroscopy reveals equilibrium protein folding dynamics and the low-force response of von Willebrand factor. Proc Natl Acad Sci U S A.

[47] Lu H, Schulten K (2000). The key event in force-induced unfolding of Titin’s immunoglobulin domains. Biophys J.

[48] Franz F, Daday C, Grater F (2020). Advances in molecular simulations of protein mechanical properties and function. Curr Opin Struct Biol.

[49] Berkovich R, Garcia-Manyes S, Urbakh M, Klafter J, Fernandez JM (2010). Collapse dynamics of single proteins extended by force. Biophys J.

[50] Smith SB, Cui Y, Bustamante C (1996). Overstretching B-DNA: the elastic response of individual double-stranded and single-stranded DNA molecules. Science.

[51] Bosco A, Camunas-Soler J, Ritort F (2014). Elastic properties and secondary structure formation of single-stranded DNA at monovalent and divalent salt conditions. Nucleic Acids Res.

[52] Dudko OK, Hummer G, Szabo A (2006). Intrinsic rates and activation free energies from single-molecule pulling experiments. Phys Rev Lett.

[53] Stannard A (2021). Molecular Fluctuations as a Ruler of Force-Induced Protein Conformations. Nano Lett.

[54] Li H, Carrion-Vazquez M, Oberhauser AF, Marszalek PE, Fernandez JM (2000). Point mutations alter the mechanical stability of immunoglobulin modules. Nat Struct Biol.

[55] Carrion-Vazquez M (2003). The mechanical stability of ubiquitin is linkage dependent. Nat Struct Biol.

[56] Brockwell DJ (2003). Pulling geometry defines the mechanical resistance of a beta-sheet protein. Nat Struct Biol.

[57] Carl P, Kwok CH, Manderson G, Speicher DW, Discher DE (2001). Forced unfolding modulated by disulfide bonds in the Ig domains of a cell adhesion molecule. Proc Natl Acad Sci U S A.

[58] Wiita AP, Ainavarapu SR, Huang HH, Fernandez JM (2006). Force-dependent chemical kinetics of disulfide bond reduction observed with single-molecule techniques. Proc Natl Acad Sci U S A.

[59] Alegre-Cebollada J, Badilla CL, Fernandez JM (2010). Isopeptide bonds block the mechanical extension of pili in pathogenic Streptococcus pyogenes. J Biol Chem.

[60] Beedle AEM, Mora M, Lynham S, Stirnemann G, Garcia-Manyes S (2017). Tailoring protein nanomechanics with chemical reactivity. Nat Commun.

[61] del Rio A (2009). Stretching single talin rod molecules activates vinculin binding. Science.

[62] Ainavarapu SR (2007). Contour length and refolding rate of a small protein controlled by engineered disulfide bonds. Biophys J.

[63] Vogel V (2006). Mechanotransduction involving multimodular proteins: converting force into biochemical signals. Annu Rev Biophys Biomol Struct.

[64] Wright CF, Teichmann SA, Clarke J, Dobson CM (2005). The importance of sequence diversity in the aggregation and evolution of proteins. Nature.

[65] Han JH, Batey S, Nickson AA, Teichmann SA, Clarke J (2007). The folding and evolution of multidomain proteins. Nat Rev Mol Cell Biol.

[66] Oberhauser AF, Marszalek PE, Carrion-Vazquez M, Fernandez JM (1999). Single protein misfolding events captured by atomic force microscopy. Nat Struct Biol.

[67] Frantz C, Stewart KM, Weaver VM (2010). The extracellular matrix at a glance. J Cell Sci.

[68] Oberhauser AF, Badilla-Fernandez C, Carrion-Vazquez M, Fernandez JM (2002). The mechanical hierarchies of fibronectin observed with single-molecule AFM. J Mol Biol.

[69] Rief M, Gautel M, Schemmel A, Gaub HE (1998). The mechanical stability of immunoglobulin and fibronectin III domains in the muscle protein titin measured by atomic force microscopy. Biophys J.

[70] Oberhauser AF, Marszalek PE, Erickson HP, Fernandez JM (1998). The molecular elasticity of the extracellular matrix protein tenascin. Nature.

[71] Rief M, Oesterhelt F, Heymann B, Gaub HE (1997). Single Molecule Force Spectroscopy on Polysaccharides by Atomic Force Microscopy. Science.

[72] Ott W (2017). Elastin-like Polypeptide Linkers for Single-Molecule Force Spectroscopy. ACS Nano.

[73] Berkovich R, Fernandez VI, Stirnemann G, Valle-Orero J, Fernandez JM (2018). Segmentation and the Entropic Elasticity of Modular Proteins. J Phys Chem Lett.

[74] Craig D, Krammer A, Schulten K, Vogel V (2001). Comparison of the early stages of forced unfolding for fibronectin type III modules. Proc Natl Acad Sci U S A.

[75] Peng Q (2009). Mechanical design of the third FnIII domain of tenascin-C. J Mol Biol.

[76] Zhuang S, Peng Q, Cao Y, Li H (2009). Modulating the mechanical stability of extracellular matrix protein tenascin-C in a controlled and reversible fashion. J Mol Biol.

[77] Ng SP (2007). Designing an extracellular matrix protein with enhanced mechanical stability. Proc Natl Acad Sci U S A.

[78] Hynes RO (2009). The extracellular matrix: not just pretty fibrils. Science.

[79] Zollinger AJ, Smith ML (2017). Fibronectin, the extracellular glue. Matrix Biol.

[80] Vogel V (2018). Unraveling the Mechanobiology of Extracellular Matrix. Annu Rev Physiol.

[81] Klotzsch E (2009). Fibronectin forms the most extensible biological fibers displaying switchable force-exposed cryptic binding sites. Proc Natl Acad Sci U S A.

[82] Gao M (2003). Structure and functional significance of mechanically unfolded fibronectin type III1 intermediates. Proc Natl Acad Sci U S A.

[83] Li H, Kong N, Laver B, Liu J (2016). Hydrogels Constructed from Engineered Proteins. Small.

[84] Li H (2021). There Is Plenty of Room in The Folded Globular Proteins: Tandem Modular Elastomeric Proteins Offer New Opportunities in Engineering Protein-Based Biomaterials. Advanced NanoBiomed Research.

[85] Arnoldini S (2017). Novel peptide probes to assess the tensional state of fibronectin fibers in cancer. Nat Commun.

[86] Schwartz MA (2010). Integrins and extracellular matrix in mechanotransduction. Cold Spring Harb Perspect Biol.

[87] Friedland JC, Lee MH, Boettiger D (2009). Mechanically activated integrin switch controls alpha5beta1 function. Science.

[88] Min D, Jefferson RE, Bowie JU, Yoon TY (2015). Mapping the energy landscape for second-stage folding of a single membrane protein. Nat Chem Biol.

[89] Zocher M (2012). Single-molecule force spectroscopy from nanodiscs: an assay to quantify folding, stability, and interactions of native membrane proteins. ACS Nano.

[90] Geiger B, Spatz JP, Bershadsky AD (2009). Environmental sensing through focal adhesions. Nat Rev Mol Cell Biol.

[91] Elosegui-Artola A, Trepat X, Roca-Cusachs P (2018). Control of Mechanotransduction by Molecular Clutch Dynamics. Trends Cell Biol.

[92] Goult BT, Brown NH, Schwartz MA (2021). Talin in mechanotransduction and mechanomemory at a glance. J Cell Sci.

[93] Goult BT, Yan J, Schwartz MA (2018). Talin as a mechanosensitive signaling hub. J Cell Biol.

[94] Haining AW, von Essen M, Attwood SJ, Hytonen VP, Del Rio Hernandez A (2016). All Subdomains of the Talin Rod Are Mechanically Vulnerable and May Contribute To Cellular Mechanosensing. ACS Nano.

[95] Yao M (2016). The mechanical response of talin. Nat Commun.

[96] Yao M (2014). Mechanical activation of vinculin binding to talin locks talin in an unfolded conformation. Sci Rep.

[97] Tapia-Rojo R, Alonso-Caballero A, Fernandez JM (2020). Talin folding as the tuning fork of cellular mechanotransduction. Proc Natl Acad Sci U S A.

[98] Gingras AR (2005). Mapping and consensus sequence identification for multiple vinculin binding sites within the talin rod. J Biol Chem.

[99] Tapia-Rojo R, Alonso-Caballero A, Fernandez JM (2020). Direct observation of a coil-to-helix contraction triggered by vinculin binding to talin. Sci Adv.

[100] Wang Y (2021). Force-Dependent Interactions between Talin and Full-Length Vinculin. J Am Chem Soc.

[101] Zacharchenko T (2016). LD Motif Recognition by Talin: Structure of the Talin-DLC1 Complex. Structure.

[102] Li G (2011). Full activity of the deleted in liver cancer 1 (DLC1) tumor suppressor depends on an LD-like motif that binds talin and focal adhesion kinase (FAK). Proc Natl Acad Sci U S A.

[103] Haining AWM (2018). Mechanotransduction in talin through the interaction of the R8 domain with DLC1. PLoS Biol.

[104] Gough RE (2021). Talin mechanosensitivity is modulated by a direct interaction with cyclin-dependent kinase-1. J Biol Chem.

[105] Mitra SK, Hanson DA, Schlaepfer DD (2005). Focal adhesion kinase: in command and control of cell motility. Nat Rev Mol Cell Biol.

[106] Seong J (2013). Distinct biophysical mechanisms of focal adhesion kinase mechanoactivation by different extracellular matrix proteins. Proc Natl Acad Sci U S A.

[107] Bell S, Terentjev EM (2017). Focal Adhesion Kinase: The Reversible Molecular Mechanosensor. Biophys J.

[108] Bauer MS (2019). Structural and mechanistic insights into mechanoactivation of focal adhesion kinase. Proc Natl Acad Sci U S A.

[109] Vera AM, Carrion-Vazquez M (2016). Direct Identification of Protein-Protein Interactions by Single-Molecule Force Spectroscopy. Angew Chem Int Ed Engl.

[110] Kim J, Zhang CZ, Zhang X, Springer TA (2010). A mechanically stabilized receptor-ligand flex-bond important in the vasculature. Nature.

[111] Le S, Yu M, Yan J (2019). Direct single-molecule quantification reveals unexpectedly high mechanical stability of vinculin-talin/alpha-catenin linkages. Sci Adv.

[112] Chen NP, Sun Z, Fassler R (2018). The Kank family proteins in adhesion dynamics. Curr Opin Cell Biol.

[113] Sun Z (2016). Kank2 activates talin, reduces force transduction across integrins and induces central adhesion formation. Nat Cell Biol.

[114] Yu M (2019). Force-Dependent Regulation of Talin-KANK1 Complex at Focal Adhesions. Nano Lett.

[115] Ladoux B, Nelson WJ, Yan J, Mege RM (2015). The mechanotransduction machinery at work at adherens junctions. Integr Biol (Camb).

[116] Hoffman BD, Yap AS (2015). Towards a Dynamic Understanding of Cadherin-Based Mechanobiology. Trends Cell Biol.

[117] Leckband DE, de Rooij J (2014). Cadherin adhesion and mechanotransduction. Annu Rev Cell Dev Biol.

[118] Niessen CM, Leckband D, Yap AS (2011). Tissue organization by cadherin adhesion molecules: dynamic molecular and cellular mechanisms of morphogenetic regulation. Physiol Rev.

[119] Shapiro L, Weis WI (2009). Structure and biochemistry of cadherins and catenins. Cold Spring Harb Perspect Biol.

[120] Harrison OJ (2010). Two-step adhesive binding by classical cadherins. Nat Struct Mol Biol.

[121] Boggon TJ (2002). C-cadherin ectodomain structure and implications for cell adhesion mechanisms. Science.

[122] Rakshit S, Zhang Y, Manibog K, Shafraz O, Sivasankar S (2012). Ideal, catch, and slip bonds in cadherin adhesion. Proc Natl Acad Sci U S A.

[123] Manibog K (2016). Molecular determinants of cadherin ideal bond formation: Conformation-dependent unbinding on a multidimensional landscape. Proc Natl Acad Sci U S A.

[124] Huber AH, Weis WI (2001). The structure of the beta-catenin/E-cadherin complex and the molecular basis of diverse ligand recognition by beta-catenin. Cell.

[125] Yamada S, Pokutta S, Drees F, Weis WI, Nelson WJ (2005). Deconstructing the cadherin-catenin-actin complex. Cell.

[126] Borghi N (2012). E-cadherin is under constitutive actomyosin-generated tension that is increased at cell-cell contacts upon externally applied stretch. Proc Natl Acad Sci U S A.

[127] Yonemura S, Wada Y, Watanabe T, Nagafuchi A, Shibata M (2010). alpha-Catenin as a tension transducer that induces adherens junction development. Nat Cell Biol.

[128] Buckley CD (2014). Cell adhesion. The minimal cadherin-catenin complex binds to actin filaments under force. Science.

[129] Le S, Yu M, Yan J (2019). Phosphorylation Reduces the Mechanical Stability of the alpha-Catenin/ beta-Catenin Complex. Angew Chem Int Ed Engl.

[130] Valbuena A, Vera AM, Oroz J, Menendez M, Carrion-Vazquez M (2012). Mechanical properties of beta-catenin revealed by single-molecule experiments. Biophys J.

[131] Pang SM, Le S, Kwiatkowski AV, Yan J (2019). Mechanical stability of alphaT-catenin and its activation by force for vinculin binding. Mol Biol Cell.

[132] Yao M (2014). Force-dependent conformational switch of alpha-catenin controls vinculin binding. Nat Commun.

[133] Crisp M (2006). Coupling of the nucleus and cytoplasm: role of the LINC complex. J Cell Biol.

[134] Nakamura F, Stossel TP, Hartwig JH (2011). The filamins: organizers of cell structure and function. Cell Adh Migr.

[135] Nakamura F, Osborn TM, Hartemink CA, Hartwig JH, Stossel TP (2007). Structural basis of filamin A functions. J Cell Biol.

[136] Furuike S, Ito T, Yamazaki M (2001). Mechanical unfolding of single filamin A (ABP-280) molecules detected by atomic force microscopy. FEBS Lett.

[137] Schwaiger I, Kardinal A, Schleicher M, Noegel AA, Rief M (2004). A mechanical unfolding intermediate in an actin-crosslinking protein. Nat Struct Mol Biol.

[138] Xu T, Lannon H, Wolf S, Nakamura F, Brujic J (2013). Domain-domain interactions in filamin A (16-23) impose a hierarchy of unfolding forces. Biophys J.

[139] Chen H (2011). Differential mechanical stability of filamin A rod segments. Biophys J.

[140] Finer JT, Simmons RM, Spudich JA (1994). Single myosin molecule mechanics: piconewton forces and nanometre steps. Nature.

[141] Chen H (2013). Mechanical perturbation of filamin A immunoglobulin repeats 20-21 reveals potential non-equilibrium mechanochemical partner binding function. Sci Rep.

[142] Rognoni L, Most T, Zoldak G, Rief M (2014). Force-dependent isomerization kinetics of a highly conserved proline switch modulates the mechanosensing region of filamin. Proc Natl Acad Sci U S A.

[143] Sengupta A, Rognoni LE, Merkel U, Zoldak G, Rief M (2021). SlyD Accelerates trans-to-cis Prolyl Isomerization in a Mechanosignaling Protein under Load. J Phys Chem B.

[144] Rognoni L, Stigler J, Pelz B, Ylanne J, Rief M (2012). Dynamic force sensing of filamin revealed in single-molecule experiments. Proc Natl Acad Sci U S A.

[145] Blanchard A, Ohanian V, Critchley D (1989). The structure and function of alpha-actinin. J Muscle Res Cell Motil.

[146] Le S (2017). Mechanotransmission and Mechanosensing of Human alpha-Actinin 1. Cell Rep.

[147] Sun HQ, Yamamoto M, Mejillano M, Yin HL (1999). Gelsolin, a multifunctional actin regulatory protein. J Biol Chem.

[148] Robinson RC (1999). Domain movement in gelsolin: a calcium-activated switch. Science.

[149] Lv C (2014). Single-molecule force spectroscopy reveals force-enhanced binding of calcium ions by gelsolin. Nat Commun.

[150] Le S, Yu M, Bershadsky A, Yan J (2020). Mechanical regulation of formin-dependent actin polymerization. Semin Cell Dev Biol.

[151] Otomo T (2005). Structural basis of actin filament nucleation and processive capping by a formin homology 2 domain. Nature.

[152] Vavylonis D, Kovar DR, O’Shaughnessy B, Pollard TD (2006). Model of formin-associated actin filament elongation. Mol Cell.

[153] Jegou A, Carlier MF, Romet-Lemonne G (2013). Formin mDia1 senses and generates mechanical forces on actin filaments. Nat Commun.

[154] Yu M (2017). mDia1 senses both force and torque during F-actin filament polymerization. Nat Commun.

[155] Yu M (2018). Effects of Mechanical Stimuli on Profilin- and Formin-Mediated Actin Polymerization. Nano Lett.

[156] Gautel M, Djinovic-Carugo K (2016). The sarcomeric cytoskeleton: from molecules to motion. J Exp Biol.

[157] Wang Z (2021). The molecular basis for sarcomere organization in vertebrate skeletal muscle. Cell.

[158] Szent-Gyorgyi AG (2004). The early history of the biochemistry of muscle contraction. J Gen Physiol.

[159] Freundt JK, Linke WA (2019). Titin as a force-generating muscle protein under regulatory control. J Appl Physiol (1985).

[160] Linke WA, Granzier H (1998). A spring tale: new facts on titin elasticity. Biophys J.

[161] Pinotsis N (2006). Evidence for a dimeric assembly of two titin/telethonin complexes induced by the telethonin C-terminus. J Struct Biol.

[162] Garcia-Manyes S, Badilla CL, Alegre-Cebollada J, Javadi Y, Fernandez JM (2012). Spontaneous dimerization of titin protein Z1Z2 domains induces strong nanomechanical anchoring. J Biol Chem.

[163] Bertz M, Wilmanns M, Rief M (2009). The titin-telethonin complex is a directed, superstable molecular bond in the muscle Z-disk. Proc Natl Acad Sci U S A.

[164] Li H, Fernandez JM (2003). Mechanical design of the first proximal Ig domain of human cardiac titin revealed by single molecule force spectroscopy. J Mol Biol.

[165] Li H (2002). Reverse engineering of the giant muscle protein titin. Nature.

[166] Anderson BR, Bogomolovas J, Labeit S, Granzier H (2013). Single molecule force spectroscopy on titin implicates immunoglobulin domain stability as a cardiac disease mechanism. J Biol Chem.

[167] Linke WA (2002). PEVK domain of titin: an entropic spring with actin-binding properties. J Struct Biol.

[168] Sarkar A, Caamano S, Fernandez JM (2005). The elasticity of individual titin PEVK exons measured by single molecule atomic force microscopy. J Biol Chem.

[169] Pang SM, Le S, Yan J (2018). Mechanical responses of the mechanosensitive unstructured domains in cardiac titin. Biol Cell.

[170] Rivas-Pardo JA (2016). Work Done by Titin Protein Folding Assists Muscle Contraction. Cell Rep.

[171] Eckels EC, Haldar S, Tapia-Rojo R, Rivas-Pardo JA, Fernandez JM (2019). The Mechanical Power of Titin Folding. Cell Rep.

[172] Yu M, Lu JH, Le S, Yan J (2021). Unexpected Low Mechanical Stability of Titin I27 Domain at Physiologically Relevant Temperature. J Phys Chem Lett.

[173] Yuan G (2017). Elasticity of the Transition State Leading to an Unexpected Mechanical Stabilization of Titin Immunoglobulin Domains. Angew Chem Int Ed Engl.

[174] Alegre-Cebollada J (2014). S-glutathionylation of cryptic cysteines enhances titin elasticity by blocking protein folding. Cell.

[175] Beedle AE, Lynham S, Garcia-Manyes S (2016). Protein S-sulfenylation is a fleeting molecular switch that regulates non-enzymatic oxidative folding. Nat Commun.

[176] Giganti D, Yan K, Badilla CL, Fernandez JM, Alegre-Cebollada J (2018). Disulfide isomerization reactions in titin immunoglobulin domains enable a mode of protein elasticity. Nat Commun.

[177] Grutzner A (2009). Modulation of titin-based stiffness by disulfide bonding in the cardiac titin N2-B unique sequence. Biophys J.

[178] Hidalgo C (2009). PKC phosphorylation of titin’s PEVK element: a novel and conserved pathway for modulating myocardial stiffness. Circ Res.

[179] Kruger M (2009). Protein kinase G modulates human myocardial passive stiffness by phosphorylation of the titin springs. Circ Res.

[180] Perkin J (2015). Phosphorylating Titin’s Cardiac N2B Element by ERK2 or CaMKIIdelta Lowers the Single Molecule and Cardiac Muscle Force. Biophys J.

[181] Zhu Y, Bogomolovas J, Labeit S, Granzier H (2009). Single molecule force spectroscopy of the cardiac titin N2B element: effects of the molecular chaperone alphaB-crystallin with disease-causing mutations. J Biol Chem.

[182] Perales-Calvo J, Giganti D, Stirnemann G, Garcia-Manyes S (2018). The force-dependent mechanism of DnaK-mediated mechanical folding. Sci Adv.

[183] Linke WA, Kruger M (2010). The giant protein titin as an integrator of myocyte signaling pathways. Physiology (Bethesda).

[184] Puchner EM (2008). Mechanoenzymatics of titin kinase. Proc Natl Acad Sci U S A.

[185] Grater F, Shen J, Jiang H, Gautel M, Grubmuller H (2005). Mechanically induced titin kinase activation studied by force-probe molecular dynamics simulations. Biophys J.

[186] Gautel M (2011). The sarcomeric cytoskeleton: who picks up the strain?. Curr Opin Cell Biol.

[187] Grison M, Merkel U, Kostan J, Djinovic-Carugo K, Rief M (2017). alpha-Actinin/titin interaction: A dynamic and mechanically stable cluster of bonds in the muscle Z-disk. Proc Natl Acad Sci U S A.

[188] Pernigo S (2010). Structural insight into M-band assembly and mechanics from the titin-obscurin-like-1 complex. Proc Natl Acad Sci U S A.

[189] Berkemeier F (2011). Fast-folding alpha-helices as reversible strain absorbers in the muscle protein myomesin. Proc Natl Acad Sci U S A.

[190] Pinotsis N (2012). Superhelical architecture of the myosin filament-linking protein myomesin with unusual elastic properties. PLoS Biol.

[191] Pernigo S (2017). Binding of Myomesin to Obscurin-Like-1 at the Muscle M-Band Provides a Strategy for Isoform-Specific Mechanical Protection. Structure.

[192] Bera M, Ainavarapu SR, Sengupta K (2016). Significance of 1B and 2B domains in modulating elastic properties of lamin A. Sci Rep.

[193] Bera M (2014). Characterization of unfolding mechanism of human lamin A Ig fold by single-molecule force spectroscopy-implications in EDMD. Biochemistry.

[194] Goldman DH (2015). Ribosome. Mechanical force releases nascent chain-mediated ribosome arrest in vitro and in vivo. Science.

[195] Aubin-Tam ME, Olivares AO, Sauer RT, Baker TA, Lang MJ (2011). Single-molecule protein unfolding and translocation by an ATP-fueled proteolytic machine. Cell.

[196] Sen M (2013). The ClpXP protease unfolds substrates using a constant rate of pulling but different gears. Cell.

[197] Olivares AO, Baker TA, Sauer RT (2018). Mechanical Protein Unfolding and Degradation. Annu Rev Physiol.

[198] Carrion-Vazquez M (1999). Mechanical and chemical unfolding of a single protein: a comparison. Proc Natl Acad Sci U S A.

[199] Popa I (2013). Nanomechanics of HaloTag Tethers. Journal of the American Chemical Society.

[200] Dietz H, Rief M (2004). Exploring the energy landscape of GFP by single-molecule mechanical experiments. Proc Natl Acad Sci U S A.

[201] Olivares AO, Kotamarthi HC, Stein BJ, Sauer RT, Baker TA (2017). Effect of directional pulling on mechanical protein degradation by ATP-dependent proteolytic machines. Proc Natl Acad Sci U S A.

[202] Johnson CP, Tang HY, Carag C, Speicher DW, Discher DE (2007). Forced unfolding of proteins within cells. Science.

[203] Krieger CC (2011). Cysteine shotgun-mass spectrometry (CS-MS) reveals dynamic sequence of protein structure changes within mutant and stressed cells. Proc Natl Acad Sci U S A.

[204] Gilbert HTJ (2019). Nuclear decoupling is part of a rapid protein-level cellular response to high-intensity mechanical loading. Nat Commun.

[205] Saini K, Discher DE (2019). Forced Unfolding of Proteins Directs Biochemical Cascades. Biochemistry.

[206] Sawada Y (2006). Force sensing by mechanical extension of the Src family kinase substrate p130Cas. Cell.

[207] Guilluy C (2014). Isolated nuclei adapt to force and reveal a mechanotransduction pathway in the nucleus. Nat Cell Biol.

[208] Elosegui-Artola A (2016). Mechanical regulation of a molecular clutch defines force transmission and transduction in response to matrix rigidity. Nat Cell Biol.

[209] Spadaro D (2017). Tension-Dependent Stretching Activates ZO-1 to Control the Junctional Localization of Its Interactors. Curr Biol.

[210] Rivas-Pardo JA (2020). A HaloTag-TEV genetic cassette for mechanical phenotyping of proteins from tissues. Nat Commun.

[211] Infante E (2019). The mechanical stability of proteins regulates their translocation rate into the cell nucleus. Nature Physics.

[212] Elosegui-Artola A (2017). Force Triggers YAP Nuclear Entry by Regulating Transport across Nuclear Pores. Cell.

[213] Randles LG, Rounsevell RW, Clarke J (2007). Spectrin domains lose cooperativity in forced unfolding. Biophys J.

[214] Gayrard C, Borghi N (2016). FRET-based Molecular Tension Microscopy. Methods.

[215] Cost AL, Ringer P, Chrostek-Grashoff A, Grashoff C (2015). How to Measure Molecular Forces in Cells: A Guide to Evaluating Genetically-Encoded FRET-Based Tension Sensors. Cell Mol Bioeng.

[216] Grashoff C (2010). Measuring mechanical tension across vinculin reveals regulation of focal adhesion dynamics. Nature.

[217] Austen K (2015). Extracellular rigidity sensing by talin isoform-specific mechanical linkages. Nat Cell Biol.

[218] Ringer P (2017). Multiplexing molecular tension sensors reveals piconewton force gradient across talin-1. Nat Methods.

[219] Arsenovic PT (2016). Nesprin-2G, a Component of the Nuclear LINC Complex, Is Subject to Myosin-Dependent Tension. Biophys J.

